# Inhibiting tau-induced elevated nSMase2 activity and ceramides is therapeutic in an Alzheimer’s disease mouse model

**DOI:** 10.1186/s40035-023-00383-9

**Published:** 2023-12-04

**Authors:** Carolyn Tallon, Benjamin J. Bell, Medhinee M. Malvankar, Pragney Deme, Carlos Nogueras-Ortiz, Erden Eren, Ajit G. Thomas, Kristen R. Hollinger, Arindom Pal, Maja Mustapic, Meixiang Huang, Kaleem Coleman, Tawnjerae R. Joe, Rana Rais, Norman J. Haughey, Dimitrios Kapogiannis, Barbara S. Slusher

**Affiliations:** 1 Johns Hopkins Drug Discovery, Johns Hopkins University School of Medicine, Baltimore, MD 21205 USA; 2 Neurology, Johns Hopkins University School of Medicine, Baltimore, MD 21205 USA; 3 Cell Biology, Johns Hopkins University School of Medicine, Baltimore, MD 21205 USA; 4 Oncology, Johns Hopkins University School of Medicine, Baltimore, MD 21205 USA; 5 Medicine, Johns Hopkins University School of Medicine, Baltimore, MD 21205 USA; 6 Pharmacology and Molecular Sciences, Johns Hopkins University School of Medicine, Baltimore, MD 21205 USA; 7 Psychiatry and Behavioral Science, Johns Hopkins University School of Medicine, 855 N. Wolfe Street, Rangos 278, Baltimore, MD 21205 USA; 8 Intramural Research Program, Laboratory of Clinical Investigation, National Institute on Aging, National Institutes of Health, 251 Bayview Blvd, Ste 8C228, Baltimore, MD 21224 USA; 9 Johns Hopkins University School of Medicine, 600 N. Wolfe Street, Pathology 517, Baltimore, MD 21287 USA

**Keywords:** Alzheimer’s disease, Extracellular vesicles, Neutral sphingomyelinase 2, Tau, Ceramide

## Abstract

**Background:**

Cognitive decline in Alzheimer’s disease (AD) is associated with hyperphosphorylated tau (pTau) propagation between neurons along synaptically connected networks, in part via extracellular vesicles (EVs). EV biogenesis is triggered by ceramide enrichment at the plasma membrane from neutral sphingomyelinase2 (nSMase2)-mediated cleavage of sphingomyelin. We report, for the first time, that human tau expression elevates brain ceramides and nSMase2 activity.

**Methods:**

To determine the therapeutic benefit of inhibiting this elevation, we evaluated PDDC, the first potent, selective, orally bioavailable, and brain-penetrable nSMase2 inhibitor in the transgenic PS19 AD mouse model. Additionally, we directly evaluated the effect of PDDC on tau propagation in a mouse model where an adeno-associated virus (AAV) encoding P301L/S320F double mutant human tau was stereotaxically-injected unilaterally into the hippocampus. The contralateral transfer of the double mutant human tau to the dentate gyrus was monitored. We examined ceramide levels, histopathological changes, and pTau content within EVs isolated from the mouse plasma.

**Results:**

Similar to human AD, the PS19 mice exhibited increased brain ceramide levels and nSMase2 activity; both were completely normalized by PDDC treatment. The PS19 mice also exhibited elevated tau immunostaining, thinning of hippocampal neuronal cell layers, increased mossy fiber synaptophysin immunostaining, and glial activation, all of which were pathologic features of human AD. PDDC treatment reduced these changes. The plasma of PDDC-treated PS19 mice had reduced levels of neuronal- and microglial-derived EVs, the former carrying lower pTau levels, compared to untreated mice. In the tau propagation model, PDDC normalized the tau-induced increase in brain ceramides and significantly reduced the amount of tau propagation to the contralateral side.

**Conclusions:**

PDDC is a first-in-class therapeutic candidate that normalizes elevated brain ceramides and nSMase2 activity, leading to the slowing of tau spread in AD mice.

**Supplementary Information:**

The online version contains supplementary material available at 10.1186/s40035-023-00383-9.

## Introduction

Alzheimer’s disease (AD) is the most common neurodegenerative disease, characterized by accumulation of amyloid plaques and tau-containing neurofibrillary tangles. Multiple therapeutic strategies are actively being pursued, with two monoclonal antibodies against amyloid-beta (Aβ), lecanemab (Leqembi®) and aducanumab (Aduhelm®), recently demonstrating ability to reduce brain amyloid plaques and delay cognitive decline, thereby earning FDA approval [1]. While encouraging, the magnitude of the therapeutic effects are moderate, so identifying additional therapeutic strategies to improve upon these initial treatments is still needed [2]. Deposits of hyperphosphorylated tau (pTau) constitute a second pathological hallmark of AD [3] and their co-occurrence with Aβ predicts cognitive decline [4]. Tau can propagate between neuroanatomically connected regions both in a free form or via extracellular vesicles (EVs) [5, 6]. Anti-tau antibodies have demonstrated the ability to sequester and degrade pTau aggregates [7], yet clinical trials to date have been unsuccessful [8, 9]. EV-associated pTau in human AD has been characterized [10] and shown to have tau-seeding capabilities [11], but the therapeutic strategy of halting the spread of tau via EV inhibition remains unexplored.

The enzyme neutral sphingomyelinase 2 (nSMase2) cleaves sphingomyelin into ceramide, which enriches in the plasma membrane enhancing membrane curvature, resulting in EV budding [12, 13]. Studies from our laboratory [14, 15] and others [16, 17] using nSMase2 genetic knock-down as well as structurally distinct small molecule inhibitors have demonstrated that inhibition of nSMase2 halts the spread of pTau. Although these data are supportive of nSMase2 inhibition as a therapeutic approach, there are no available inhibitors suitable for clinical development. Employing high-throughput screening followed by extensive medicinal chemistry [18, 19], our lab identified PDDC, the first nM potent, orally bioavailable, and brain-penetrable small molecule as an inhibitor of nSMase2. We previously demonstrated that PDDC effectively reduced the plasma levels of brain-derived EVs following acute brain injury [20] and exhibited cognitive benefit in the 5 × FAD amyloid mouse model of AD [19]. Here, we build upon these results by focusing on the other pathological hallmark of AD, tau, and evaluate PDDC’s potential to attenuate tau propagation and disease progression in PS19 tau transgenic mice as well as in an AAV-mediated human tau propagation model.

## Materials and methods

### Study design

This study examined the effect of tau expression on nSMase2 enzymatic activity and ceramide production as well as the therapeutic efficacy of the nSMase2 inhibitor PDDC in mouse models of AD. We utilized two distinct AD mouse models: PS19 transgenic mice and an AAV-hTau (P301L/S320F) propagation model. Animals were randomly assigned to either vehicle or drug group with an equal number of male and female in each. All data were acquired in a blinded manner with a number assigned to each animal unrelated to their treatment status. Where possible, the experimenter dosing the animals was different from the experimenter carrying out the data acquisition and statistical analysis to maintain the blind. Sample sizes were determined using power calculations where the averages, differences between groups, and standard deviations were based on previously observed statistically significant differences in similar experiments to generate at least 90% power with five extra animals per group to account for any premature animal losses. Cell culture experiments were run twice in duplicate and animal studies were run with two separate cohorts of equal sizes for reproducibility.

### Primary neuronal cell culture

Primary hippocampal neurons were isolated from day-18 embryos of Sprague–Dawley rats acquired from Jackson Laboratories (Bar Harbor, ME), as previously described [30–32]. Briefly, the hippocampal tissue was dissected, gently titurated with trypsinization in calcium-free Hank’s balanced salt solution (HBSS, free of calcium, magnesium, and phenol red; Corning Inc., Manassass, VA) and cells were resuspended in Neurobasal media (Gibco, Waltham, MA) supplemented with B27 (Gibco), 1% antibiotic/antimitotic solution (Gibco), 10% FBS (Sigma, St. Louis, MO), HEPES (4.8 mM, Sigma) and *L*-glutamine (1.2 mM, Sigma). For imaging studies, cells were plated on 12-mm glass coverslips coated with polyethyleneimine (PEI, Sigma) at a density of 70,000 cells/well. For nSMase2 activity and ceramide analyses, cells were directly plated on PEI-coated 12-well cell culture plates at a density of 700,000 cells/well. After 4 days in vitro (DIV), cells were transduced with either pAAV1-CAG-GFP (Addgene, Watertown, MA) or AAV1-serotype viral particles packaged with the hTau vector CBA-hTau24(P301L)(S320F)-WPRE (kindly provided by the Chakrabarty lab, University of Florida) [33]. Cells were transduced at a multiplicity of infection of 50,000. At 8 DIV, cells were harvested for nSMase2 activity and ceramide assessments or prepared for imaging. Cells for nSMase2 activity assessment were washed twice with ice-cold DMEM/F12 containing HEPES and without phenol red (ThermoFisher,Waltham, MA) and incubated with mammalian protein extraction reagent (ThermoFisher) containing 1 × HALT protease inhibitor without EDTA (ThermoFisher), with shaking for 10 min at 4 °C and for another 5 min at room temperature (RT). Final cell detachment was carried out using cell scrapers and cells were sonicated (three 15-s pulses on ice). The resulting lysates were assayed for both nSMase2 activity and total protein content as detailed below. Cells harvested for ceramide assessments were washed 3 times on ice with 1 × PBS prior to gentle scraping with a cell scraper, and centrifuged at 300 × *g* for 5 min at 4 °C. The pellet was frozen in liquid nitrogen, and stored at − 80 °C. Cells for imaging were fixed with ice-cold 4% paraformaldehyde (PFA, Electron Microscopy Sciences) for 10 min and washed 3 times in 1 × PBS followed by immediate staining.

### Animal studies and PDDC dosing

All animal care and experimental procedures complied with the National Institutes of Health guidelines on animal care and were approved by the Johns Hopkins University Institutional Animal Care and Use Committee. Mice were housed in a temperature- and humidity‐controlled environment under a 14‐h light:10-h dark cycle. Food and water were available ad libitum. Animals were acclimated to the facility for at least 7 days after arrival, prior to any experimentation. PDDC was synthesized in our laboratory as previously described [18, 19], and formulated into an OpenStandard Diet (15 kcal% mouse chow) at an approximate 100 mg/kg daily dose. The animals were treated as previously described [20]. PS19 breeder mice were purchased from Jackson Laboratories (strain #008169) and bred in-house to generate appropriately sized litters. Non-carrier littermates were deemed WT controls. Dosing for PS19 animals was initiated at 4 months of age, prior to overt pathology and symptom onset [34], and continued till the age of 9 months. For the AAV-hTau mice, C57BL6/J mice were purchased from Jackson Labs (strain #000664) and stereotaxically injected at 10 weeks of age and then dosed for 6 weeks. Body weights were measured weekly for all studies. Equal numbers of male and female animals were enrolled. No significant differences between males and females were observed, so the groups were combined.

At the end of the study, animals were euthanized by an overdose of isoflurane. The chest cavity was opened up and blood was collected via cardiac puncture into cold EDTA‐coated BD microtainers (Franklin Lakes, NJ). For histological assessments, the animals were transcardially perfused with 1 × ice-cold PBS followed by 2% PFA (Electron Microscopy Sciences). For all other experiments, mice were transcardially perfused with PBS only. Tissue was collected following perfusion and the assays were performed as described below.

### PDDC in vivo pharmacokinetics (PK) and bioanalysis

Twelve mice (6 males and 6 females) were enrolled in a PK study. The brain and plasma levels of PDDC were quantified at 4 time points throughout the 24-h day (00:00, 07:00, 12:00, and 19:00), with *n* = 3 at each time point. The time points were selected based on the 14-h light and 10-h dark cycle with light on at 07:00 and light off 2 h after 19:00. After 4-week PDDC-containing chow, whole blood was collected via cardiac puncture and plasma was isolated by centrifugation at 500 × *g* for 15 min and stored at − 80 °C until LC/MS/MS bioanalysis. Whole brains were harvested following blood collection and cut into hemispheres before freezing in liquid nitrogen and stored at − 80 °C.

The bioanalysis was carried out as previously described [18, 20]. Briefly, protein precipitation using acetonitrile (Sigma-Aldrich, St. Louis, MO)(100% *v*/*v*) containing the internal standard (losartan 500 nM; Tocris, Minneapolis, MN) was used to extract PDDC standards and samples from plasma and brain, followed by vortexing and centrifugation at 10,000 × *g*. The supernatant was diluted 1:1 with water and then analyzed via LC/MS/MS. Plasma (nmol/ml) and tissue (nmol/g) concentrations were determined and plots of mean plasma concentration versus time were constructed. Phoenix WinNonlin version 7.0 (Certara USA, Inc., Princeton, NJ) was used to quantify exposure (AUC_0–t_) using non‐compartmental analysis modules.

### nSMase2 enzymatic activity assay

nSMase2 enzymatic activity was assessed as previously described [35]. nSMase2 activity measurement was initiated upon the addition of sphingomyelin (20 µM) to produce ceramide and phosphorylcholine, the latter of which was dephosphorylated by the coupling enzyme alkaline phosphatase (4 U/ml), producing choline. The choline was then oxidized by choline oxidase (0.1 U/ml) to generate betaine and H_2_O_2_, which produces resorufin, a fluorescent molecule, in the presence of horseradish peroxidase (1 U/ml) and Amplex Red (25 µl). The reactions were performed in a total reaction volume of 50 µl in 384-well microplates for 3 h at 37 °C in 100 mM Tris–HCl pH 7.4, 10 mM MgCl2, and 0.2% Triton X-100. At the end of the reaction period, the relative fluorescence units (RFUs) were measured with excitation at 530 nm and emission at 590 nm. Total protein measurements were carried out as per manufacturer’s instructions using the BioRad Detergent Compatible Protein Assay kit. Data of the enzymatic activity are presented in unit RFU/mg/h.

### Brain lipid extraction and LC–ESI–MS/MS ceramide quantification

Lipid extractions from hippocampal cells and a single-hemisphere brain tissue (for PS19) or micro-dissected hippocampi and cortex (for AAV-hTau) were carried out using a modified Bligh and Dyer method [36] as previously described [37]. Tissue samples were weighed and homogenized in water (10×) before adding 3 × methanol containing a 1.3-μg/ml internal standard of ceramide (d18:1/12:0) (Avanti Polar Lipids, Alabaster, AL) [38] followed by an addition of 4 × chloroform. Organic layers containing crude lipid extracts were collected following clear phase separation, before being dried in a nitrogen evaporator (Organomation, Berlin, MA) and stored at − 80 °C. Prior to analysis, pure methanol was used to resuspend the dried extracts. A Shimadzu ultra-fast liquid chromatography system (Shimadzu, Nakagyo-ku, Kyoto, Japan) coupled to a hybrid triple quadrupole LIT (linear ion trap) mass spectrometer 4000 QTRAP system equipped with Turbo Ion Spray (SCIEX, Foster City, CA) with an ULTRA HPLC In-Line Filter (0.5 µm Depth Filter × 0.004 in ID*)(*Phenomenex, Torrance, CA) was used to separate ceramides on a C18 reverse-phase column (particle size 2.6 µm, diameter 2.1 mm, length 50 mm). The lipids were ionized using positive electrospray ionization (ESI, +ve) and individual ceramide species were detected by multiple reaction monitoring with instrument conditions and HPLC parameters previously described [39]. Quality control samples were injected in every 10 injections. Eight-point calibration curves (0.1–1000 ng/ml) were constructed by plotting area under the curve (AUC) for each ceramide calibration standard d18:1/C16:0, d18:1/C18:0, d18:1/C20:0, d18:1/C22:0, d18:1/C24:0 (Avanti Polar Lipids, Alabaster, AL) with correlation coefficients > 0.999. Identified ceramide concentrations were calculated by fitting them to these standard curves based on acyl chain length. Instrument control and data acquisition were performed by using Analyst (version 1.4.2, SCIEX Inc. Thornhill, ON, Canada) and data analysis was completed using MultiQuant software (version 2.0, SCIEX).

### Hippocampal protein isolation and western blotting for tau

The left and right hippocampi were micro-dissected from whole brains following PBS perfusion, weighed, and then snap frozen on dry ice. The hippocampal tissue was mechanically homogenized in 10 volumes of ice-cold 1 × RIPA buffer (Thermo Fisher Scientific) with Pierce Protease and Phosphatase Inhibitor Mini Tablets (Thermo Fisher Scientific) followed by brief sonication. The lysate was centrifuged for 15 min at 10,000 × *g* at 4 °C and the supernatant was collected, frozen on dry ice, and stored at − 80 °C.

Sarkosyl-soluble and -insoluble isolation was carried out based on a modified version of Sahara and Kimura [40]. Briefly, the hippocampal tissue was mechanically homogenized in 10 volumes of ice-cold 2 × TBS buffer (Thermo Fisher Scientific) with protease and phosphatase inhibitor cocktail, followed by centrifugation at 27,000 × *g* at 4 °C for 20 min. The pellet was resuspended in five volumes of ice-cold high-salt/sucrose buffer and centrifuged at 27,000 × *g* at 4 °C for 20 min. The supernatant was then adjusted to 1% sarkosyl and incubated on a shaker for 1 h at 37 °C, followed by ultracentrifugation at 150,000 × *g* at 4 °C for 1 h. The sarkosyl-soluble supernatant was removed and frozen on dry ice to be stored at − 80 °C. The sarkosyl-insoluble pellet was resuspended in 0.5 volume of 1 × TE buffer (Thermo Fisher Scientific) and frozen on dry ice to be stored at − 80 °C.

All western blots were run in a similar manner. Equal volumes of samples were loaded onto a NuPAGE 4%–12% bis–tris protein gel (Invitrogen) and transferred onto a PVDF membrane using an iBlot2 Gel Transfer Device (Life Technologies). Total protein stain was performed for loading control using Revert 700 total protein stain (LI-COR). An HRP-conjugated GAPDH antibody was also used as a loading control where applicable. Blots were blocked with EveryBlot blocking buffer (Bio-Rad) and stained overnight at 4 °C for total tau using an antibody that binds all tau isoforms and is considered phosphorylation-independent (Tau 46; Santa Cruz Biotechnology, #sc-32274) and for Thr181 phosphotau (D9F4G; Cell Signaling Technologies, #12885S). Appropriate HRP-conjugated secondary antibodies were used. Blots were incubated briefly with Clarity ECL substrate (Bio-Rad). All blots were imaged using the Bio-Rad ChemiDoc MP imager. Analysis was done using raw TIFF files in ImageJ. Mean pixel intensity was measured for each band and normalized to the total protein or GAPDH intensity levels. To compare across blots, each blot was also normalized to the average value of the vehicle intensity.

### Immunofluorescence staining

For fixed primary neuronal cells, permeabilization was performed with 0.1% Triton X-100 in 1 × PBS (0.1% PBST) for 10 min at RT, followed by blocking with 5% normal goat serum in 0.1% PBST for 1 h at RT. Then the cells were incubated with primary antibody to phosphor-tau Thr181 (pThr181-Tau, Cell Signaling Technology) overnight at 4 °C followed by the appropriate secondary antibody for 1 h at RT. Neurons were stained using Alexa Fluor® 647-conjugated anti-NeuN antibody (Abcam, #ab190565) for 2 h at RT. Nuclei were then stained with Hoechst 33342 (Invitrogen, #H3570) before mounting with ProLong Glass antifade mountant (Invitrogen).

Brain tissues were prepped for immunofluorescence staining as previously described [15, 41]. Briefly, following PFA perfusion, whole brains were dissected out and post-fixed overnight at 4 °C in 2% PFA before being transferred to 15% then 30% sucrose, each overnight at 4 °C. Brains were then frozen in TissueTek O.C.T. (Sakura FineTek USA, Inc., Torrence, CA) and sectioned on a cryostat (Microm HM 505E, International Medical Equipment, MI) at 20-µm thickness. Sections were permeabilized and blocked followed by primary antibody (pThr181-Tau, Cell Signaling Technology; GFAP, Abcam, #ab4674; Iba1, Fujifilm Wako Chemicals, #019-19741; Synaptophysin, SinoBiological, #100298-T40) incubation overnight at 4 °C. Sections were then stained with appropriate secondary antibodies for 1 h at RT. Neurons were then stained with Alexa Fluor® 647-conjugated Anti-NeuN antibody (Abcam, #ab190565) for 2 h at RT. Nuclei were stained with Hoechst 33342 (Invitrogen, #H3570) before coverslipping with ProLong Glass antifade mountant. All slides grouped together for mean fluorescence intensity (MFI) assessments were stained in the same batch to minimize possible differences in antibody amounts and incubation times.

All images were taken with a LSM 800 confocal microscope (Zeiss) using identical imaging parameters. Images were acquired by focusing on the center of the section where the signal of interest was at a maximal intensity, and the brightest slide was used to set the imaging parameters. For each animal, 3–5 hippocampal sections from each hemisphere were imaged and 3–6 images were acquired per hippocampal section (CA1, CA3, and DG, where applicable). Values from the images of both hippocampi per section were averaged and reported. All image analyses were done using the Zen Blue imaging software (Zeiss).

### Single-cell MFI quantification

Single-cell MFI was determined using images stained with pThr181-Tau, NeuN, and Hoechst 33342 and imaged with a 40 × objective. Tau-positive neurons were determined based on triple staining of tau, NeuN, and nuclei in order to ensure cells counted were imaged at a similar level and differences in intensity were not due to different imaging planes. Cells deemed “tau+” then had their cell bodies intricately traced, stopping at the axonal hilus as it was not possible to include axons and dendrites in the tracing. The MFI of the tau signal was recorded for each cell. The MFI of all the cells quantified per section were averaged and these values were used for statistical analyses.

### Pyramidal and granular cell layer thickness

Cell layer thickness was determined using images stained with NeuN and Hoechst 33342 with a 40× objective as previously reported [42]. The thickness of the NeuN cell layer in the CA1 and DG regions was determined by drawing a line perpendicular to the cell layer at three points along the layer in each image, taking the thickest, thinnest and middle of the section. The three values were then averaged and the values for each section were then averaged and reported as a replicate. The CA3 region was not evaluated as it was more subject to slight variations in the plane of section and was highly variable.

### Synaptophysin fluorescence intensity quantification

To quantify synaptic loss previously observed in PS19 mice [34], we measured the MFI intensity of synaptophysin staining in the Mossy fiber layer of the CA3. Images were acquired with identical parameters using a 20 × objective and three images per hippocampus were taken from three sections per mouse. The Mossy fiber layer was traced and the MFI was recorded from each image. The values of all images per section were averaged and reported as a replicate.

### Iba1 and GFAP intensity quantification

Sections were stained with Iba1, GFAP, and Hoechst 33342 and imaged with a 20 × objective with identical parameters. Eight images per section from 3 sections per animal were obtained with identical parameters in the stratum radiatum, stratum moleculaire, and hilus regions around the CA1, CA3 and dentate gyrus regions. The MFI of the entire field of view was recorded and the average of all images per section was reported as a replicate.

### Plasma neuronal-derived EVs (nEV) isolation, quantification, and characterization

Mouse plasma nEV isolation was carried out as previously described [43]. Briefly, plasma samples (approximately 100 µl) were defibrinated with thrombin (System Biosciences, Mountainview, CA) for 30 min at RT and total EVs isolated via size exclusion chromatography (SmartSEC, System Biosciences). The nEVs  were isolated from the total EVs via immunocapture against L1CAM/CD171 (clone 5G3). Protease and phosphatase inhibitors were included in multiple steps. Intact EVs were used for determination of particle concentration and diameter using nanoparticle tracking analysis (NTA) (Nanosight NS500; Malvern, Amesbury, UK). To confirm the isolation of EVs via L1CAM immunocapture, 30 µl of intact L1CAM^+^ nEVs were subjected to ExoView™ for the fluorescent detection of canonical EV markers CD9, CD63 and CD81 (NanoView Biosciences, #EV-TETRA-M2). Additionally, nEVs were lysed with protein extraction solution and the protein concentration was determined using the Bradford protein assay (Bio-Rad, Hercules, CA). The pThr181-tau content was quantified in duplicate using the Human Tau pT181 ProQuantum Immunoassay Kit as per the manufacturer’s protocol (Invitrogen) at a dilution of 1:4. Samples were read on a qPCR equipment (StepOnePlus, Applied Byosystems). All Ct values were below 35. The limit of detection (LOD) was found to be 0.0847 pg/ml. To avoid excluding WT samples from the analysis, since they lack human tau, samples that read below the LOD were set at a value of ½ LOD.

Nanoscale multiplex flow cytometry analysis was carried out as previously described with slight modifications [44]. Intact L1CAM^+^ nEVs or total EVs isolated via SEC were diluted to 350 µl with 1 × PBS and incubated with an equal volume of 40 µM blue succinimidyl ester (BSE; Thermo Fisher Scientific, #C34568) for 90 min at 37 °C. Excess BSE was removed via ultrafiltration (100-kDa cutoff; Millipore Sigma, #C7719), bringing the final retentate volume to 1 ml with 1 × PBS incubated with 100 µl of Capto Core 400 beads (Cytiva, #17372401) for 30 min at RT with gentle rotation mixing. The EV supernatant was then incubated with a mouse-specific Fc receptor blocker reagent (Miltenyi, #130-092-575) for 30 min at RT with gentle rotation and labeled with fluorescent antibodies (each at 0.2 ng/µl; PE-anti-Syntenin-1, Abcam, #210837; PE-anti-pSer262-Tau, Thermo Fisher Scientific, #44-750G; APC-anti-β-III-tubulin, Biolegend, #801219; APC-Iba-1, Abcam, #5076) in 0.05% Tween-20 for partial membrane permeabilization. Labeled EVs were detected with a CytoFLEX LX flow cytometer (Beckman Coulter) using 405 nm fluorescence triggering and analyzed with CytExpert software v2.3.0.84 (Beckman Coulter). For fluorescence detection, we used a 660/10 bandpass filter for APC, and 585/42 for PE, with gain voltage not exceeding 1500 V. The instrument was aligned using FITC-tagged beads with sizes ranging from 100 to 1300 nm (100 nm beads, #834, Bangs Laboratories; 130–1300 nm beads, #NFPPS-52-4K and #NFPPS-0152-5, Spherotech). Samples were diluted with 1 × PBS to control the abort rate below 1% without exceeding the 200 events/second rate to avoid coincident detection of events, and analyzed for 3 min.

### AAV-hTau(P301L/S320F) stereotaxic injection model

The rapid tau propagation model was established as previously described [15]. The same AAV-hTau vector used in the cell culture experiments was used for animal injection. Using a modified stereotaxic surgical method [16, 33], 5 × 10^9^ viral particles in < 250 nl PBS were injected in the left dorsal hippocampus near the CA3 region with a stereotaxic apparatus (Stoelting, Wood Dale, IL) and a pulled glass capillary needle (tip diameter < 50 µm) at the coordinates AP-2.3, ML-2.1, and DV-2.2. The injection was made with a digital nanoinjector (Stoelting) attached to a mineral oil-filled 5-μl gas-tight syringe (Hamilton) over 5 min and the syringe was left in place for an additional 5 min. Afterwards, the syringe was removed and the incision closed with cyanoacrylate glue (Vetbond, 3 M). The mice were provided with ketoprofen analgesia and monitored for distress over 48 h. They were given 2 days of recovery prior to treatment initiation and were treated for 6 weeks.

### Contralateral tau MFI quantification

Sections from the AAV-hTau mice were analyzed as previously described [15]. Following staining for NeuN, phosphor-tau, and Hoechst 33342, two images from a section containing both the left and the right dorsal dentate gyrus were acquired on an LSM 800 confocal microscope (Zeiss). The two images per section were averaged and this averaged value was treated as a replicate for statistical analyses. The ratio of the contralateral to ipsilateral MFI in each section was calculated to account for injection variability. Animals with improper injection sites were excluded from analysis.

### Statistical analysis

All statistical analyses were done using GraphPad Prism 9 (GraphPad Software, LLC, San Diego, CA). Comparisons between two normally distributed groups were made with a two-tailed, unpaired student’s *t*-test. Comparisons between two non-parametric groups were made with a Mann–Whitney U test. Comparisons between three or more groups were made with one-way ANOVA with Tukey’s multiple comparisons. *P* < 0.05 was considered as statistically significant.

## Results

### Mutant tau expression in cultured neurons increases nSMase2 activity and ceramide levels

**Fig. 1:**
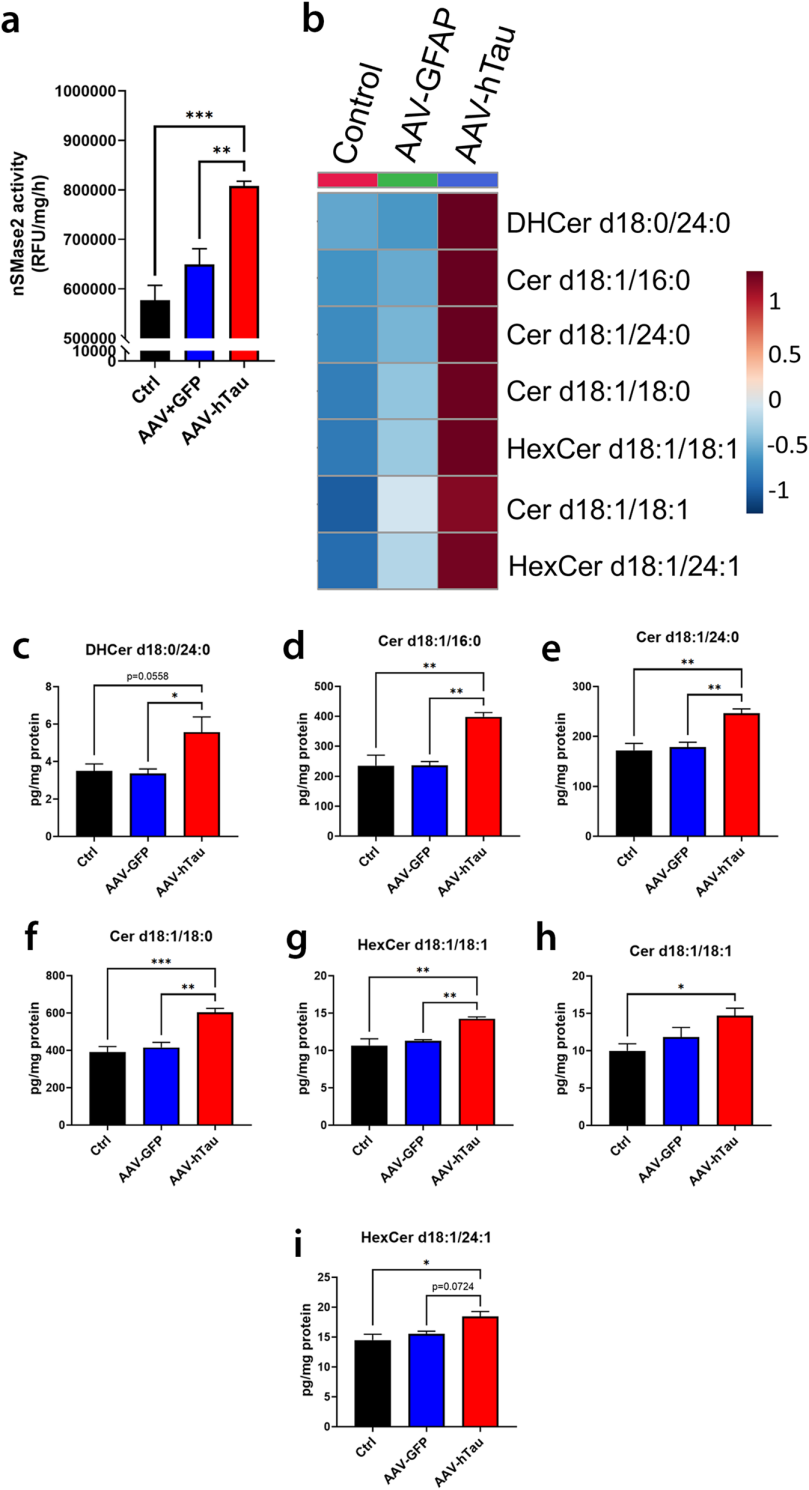
Mutant tau expression induces significant increases in nSMase2 activity and ceramide levels in cultured neurons. **a** nSMase2 activity from untransduced control (Ctrl), AAV-GFP-transduced, and AAV-hTau (P301L/S320F)-transduced cells. **b** Heat map of the significantly elevated ceramide species in AAV-hTau (P301L/S320F)-transduced cells compared to either control or AAV-GFP-transduced cells. Colors represent fold changes of relative abundance compared to untransduced control cell levels. Red indicates increased fold-change, blue represents decreased fold-change. **c–i** Individual levels of the altered ceramides. *n* = 4/group. Bars represent mean ± SEM. *P < 0.05, **P < 0.01, ***P < 0.001. One-way ANOVA with Tukey’s multiple comparison

We examined the effect of mutant tau expression in neurons on nSMase2 activity and ceramides in primary rat hippocampal neurons. We observed GFP expression in AAV-GFP-transduced cells and pTau-Thr181 staining in AAV-hTau-transduced cells (Additional file 1: Fig. S1). No fluorescent signal was observed in the untransduced controls (Additional file 1: Fig. S1). The AAV-hTau cells exhibited significantly elevated nSMase2 activity compared with untransduced controls (*P* = 0.00036) and AAV-GFP controls (*P* = 0.0048) (Fig. 1a). Of the 22 ceramide species detected, 7 were found to be significantly elevated in AAV-hTau-transduced cells compared to untransduced and/or AAV-GFP-transduced cells (Fig. 1b–i; Additional file 1: Table S1). All other forms of ceramide remained unchanged.

### Oral PDDC provides sustained brain levels of the drug and inhibits nSMase2 activity and ceramide levels

**Fig. 2:**
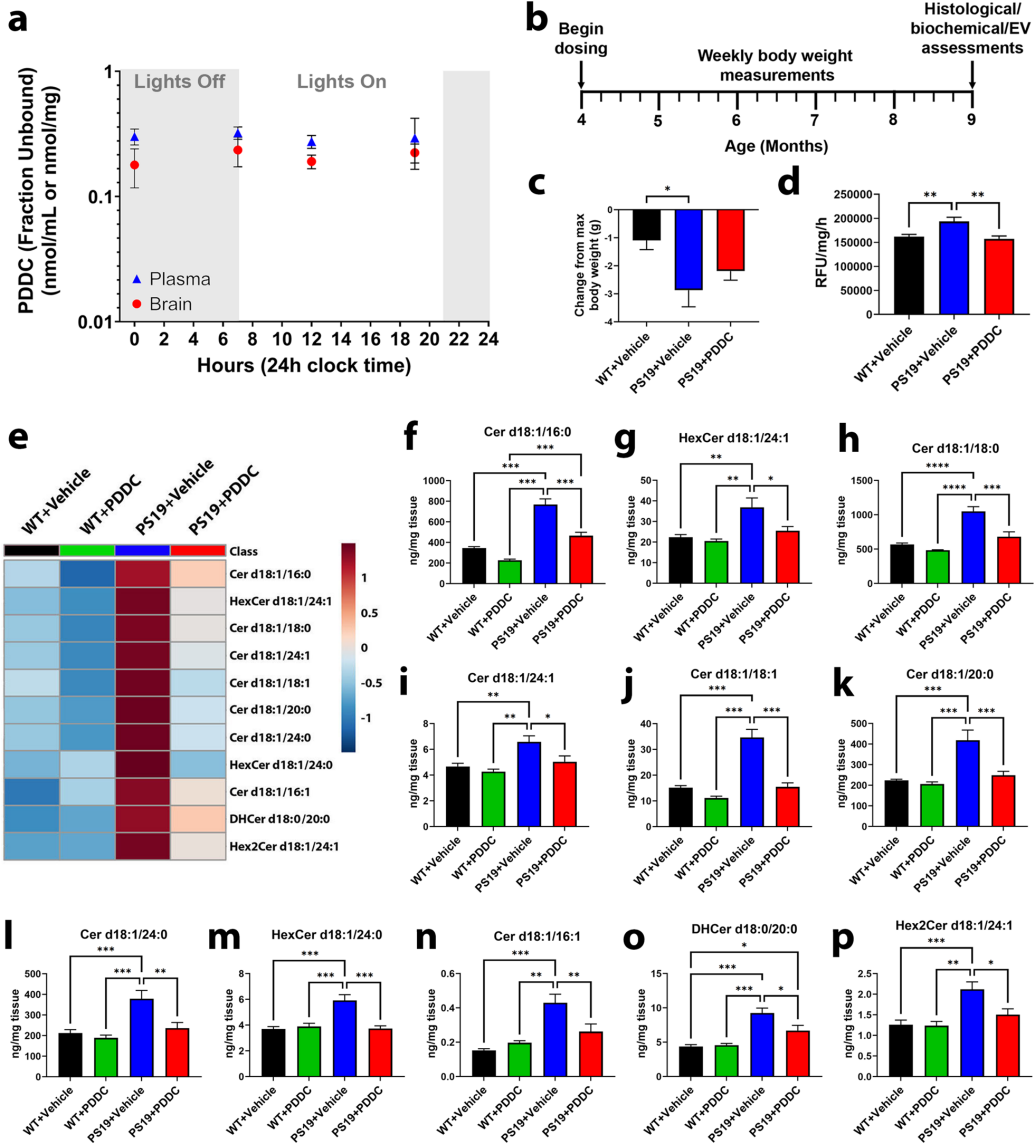
Brain ceramides are robustly elevated in PS19 mice and are normalized with PDDC treatment. **a** Plasma and brain levels of PDDC measured over 24 h following 4 weeks of dosing. *N* = 3/group at each time point. Points represent mean ± SEM. **b** Dosing schematic. **c** Percent change of body weight at the time of sacrifice from the maximum body weight over a 5-month dosing period in the WT + Vehicle, PS19 + Vehicle, and PS19 + PDDC groups. *N* = 16–20. **d** Quantification of hippocampal nSMase2 activity in the WT + Vehicle, PS19 + Vehicle, and PS19 + PDDC mice. *N* = 8–10/group. **e** Heatmap showing the ceramide species significantly reduced in PDDC-treated PS19 mice compared to vehicle-treated PS19 mice (*P* < 0.05). Colors represent relative abundance of each ceramide. **f–p** Cortical ceramide levels in WT and PS19 mice chronically treated with vehicle or PDDC. *N* = 6–11/group. Bars represent mean ± SEM. *P < 0.05. **P < 0.01. ***P < 0.001. One-way ANOVA with Tukey’s multiple comparison

We next examined the effect of PDDC on brain nSMase2 activity and ceramide levels in PS19 mice. After 4 weeks on PDDC-containing chow (approximately 3-mg PDDC ingested daily), PS19 mice exhibited sustained plasma and brain levels of PDDC at free concentrations (fraction unbound) around the IC_50_ of PDDC for nSMase2 (300 nM) (Fig. 2a). PDDC chow was fed 5 days/week to PS19 mice starting at 4 months of age and continued until 9 months of age (Fig. 2b). After 5 months of treatment, relative to their peak weight, the vehicle-treated PS19 mice lost more weight compared to the vehicle-treated WT controls (Fig. 2c, *P* = 0.018). The PDDC-treated PS19 mice showed a trend of reduced weight loss; however, the reduction was not significantly different from the vehicle-treated PS19 mice (Fig. 2c). Additionally, modified SHIRPA test did not reveal abnormalities of physical characteristics and behaviors with PDDC treatment (Additional file 1: Table S2). In the open field test, both PDDC- and vehicle-treated PS19 mice showed more ambulatory movement versus WT mice, as has been previously described [45]. There were no differences in the overall distance travelled, fine movement, rearing, or center/periphery ratio among all groups (Additional file 1: Fig. S2). In all experimental groups, the plasma clinical chemistry parameters that assess liver and kidney toxicity were within the normal ranges observed in our facility and reported by Jackson Laboratories [46–48], Charles River [49], or Taconic [50] (Additional file 1: Table S3).

Similar to cells transduced with AAV-hTau, we found that the hippocampal nSMase2 activity in the PS19 mice was significantly elevated compared to the WT mice at 9 months of age (Fig. 2d, *P* = 0.0057). PDDC treatment completely normalized this elevation, demonstrating clear target engagement (Fig. 2d, *P* = 0.003 vs. PS19 + vehicle group). Of the 46 different forms of ceramide detected, 11 were increased in the vehicle-treated PS19 mice compared to the vehicle-treated WT mice (Fig. 2e–p; Additional file 1: Table S4, *P* values found in table), and they were all reduced with PDDC treatment to levels comparable to the WT + vehicle concentrations. PDDC treatment did not alter ceramide levels in WT mice.

### PDDC treatment reduces tau pathology in PS19 mice

**Fig. 3:**
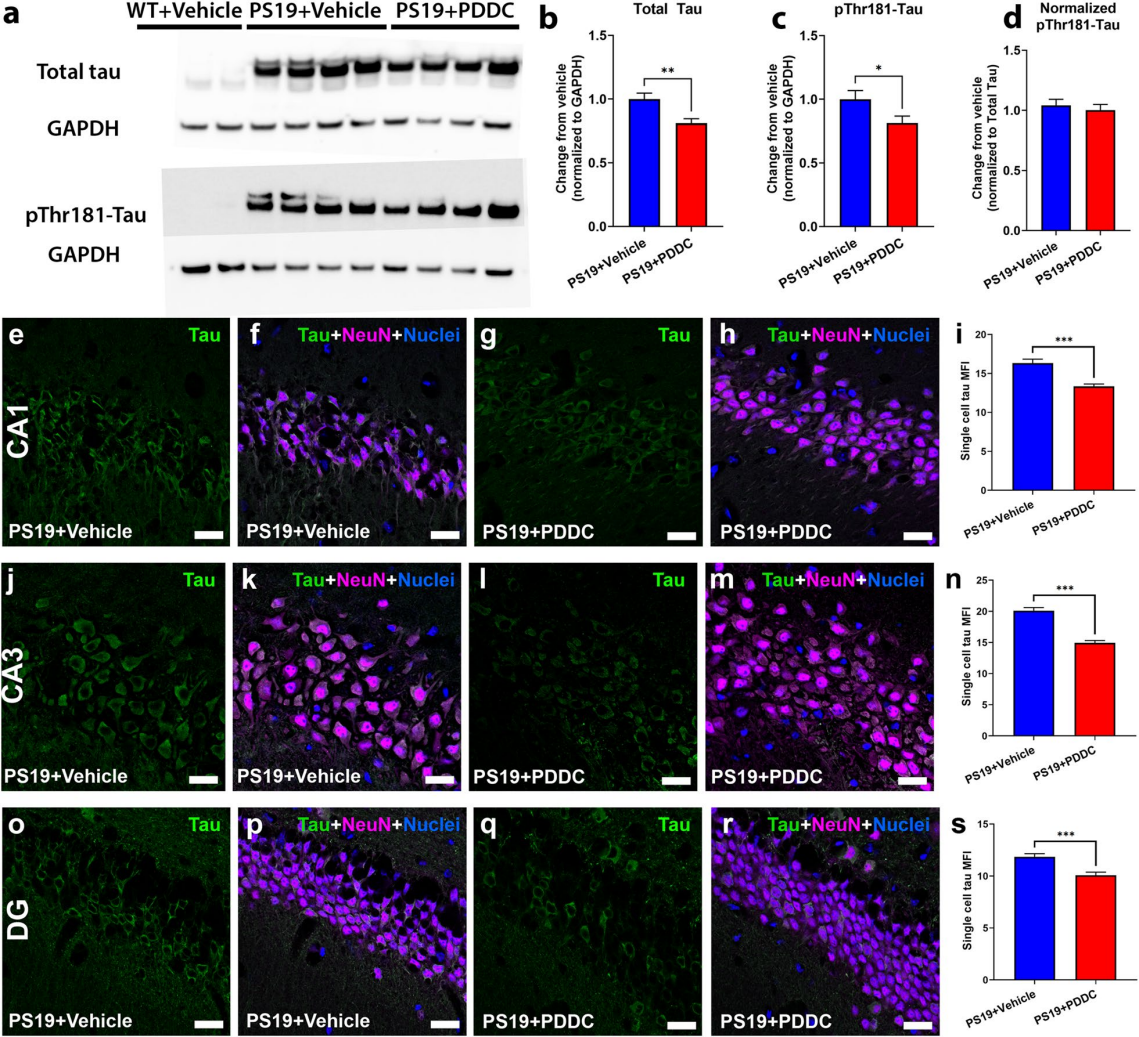
PDDC treatment reduces hippocampal tau levels in PS19 mice. **a** Representative Western blots from micro-dissected hippocampal tissue showing total human tau (upper blot) and pThr181-Tau (lower blot). GAPDH shown as a loading control. **b** Quantification of Western blots for total tau. **c** Quantification of Western blots for pThr181-Tau. **d** pThr181-Tau level normalized to total tau. *N* = 11–12/group. **e**–**s** Representative images showing pThr181-Tau staining (green) and neuronal staining (magenta) from vehicle- and PDDC-treated PS19 mice in the CA1 (**e**–**h**), CA3 (**j**–**m**) and dentate gyrus (DG, **o**–**r**). Single-cell mean fluorescence intensity (MFI) from the CA1 (**i**), CA3 (**n**), and DG (**s**). Nuclei shown in blue. *N* = 120 cells from 4 mice/group. Bars represent mean ± SEM. *P < 0.05, **P < 0.01, ***P < 0.001. Scale bars, 20 µm. Gamma and brightness adjusted equally for all images presented. All graphs, unpaired two-tailed *t*-test

We next assessed whether PDDC treatment could alter tau pathology in PS19 mice. The total tau in the hippocampus of PDDC-treated PS19 mice was reduced compared with the vehicle-treated PS19 mice (Fig. 3a, b, *P* = 0.0041). No human tau was detected in WT animals (Fig. 3a). Although pThr181-Tau was also reduced in PDDC-treated mice (Fig. 3c, *P* = 0.047), this difference was lost after total tau normalization (Fig. 3d), suggesting that PDDC does not impact the phosphorylation of tau at Thr181. PDDC also did not affect the balance of Sarkosyl-soluble and -insoluble tau fractions (Additional file 1: Figs. S3 and S4**)**. We further assessed tau levels by quantifying single-cell tau fluorescence intensity in the hippocampus (Fig. 3e–s). Tau fluorescence intensity in individual neurons was reduced in the CA1 (Fig. 3e–i, *P* = 9.56 × 10^–7^), CA3 (Fig. 3j–n, *P* = 3.49 × 10^−10^), and DG (Fig. 3o–s, *P* = 1.57 × 10^−4^) regions of the hippocampus of PDDC-treated PS19 mice compared with the vehicle-treated PS19 mice.

### PDDC treatment reduces hippocampal cell layer thinning and mossy fiber synaptophysin loss in PS19 mice

**Fig. 4:**
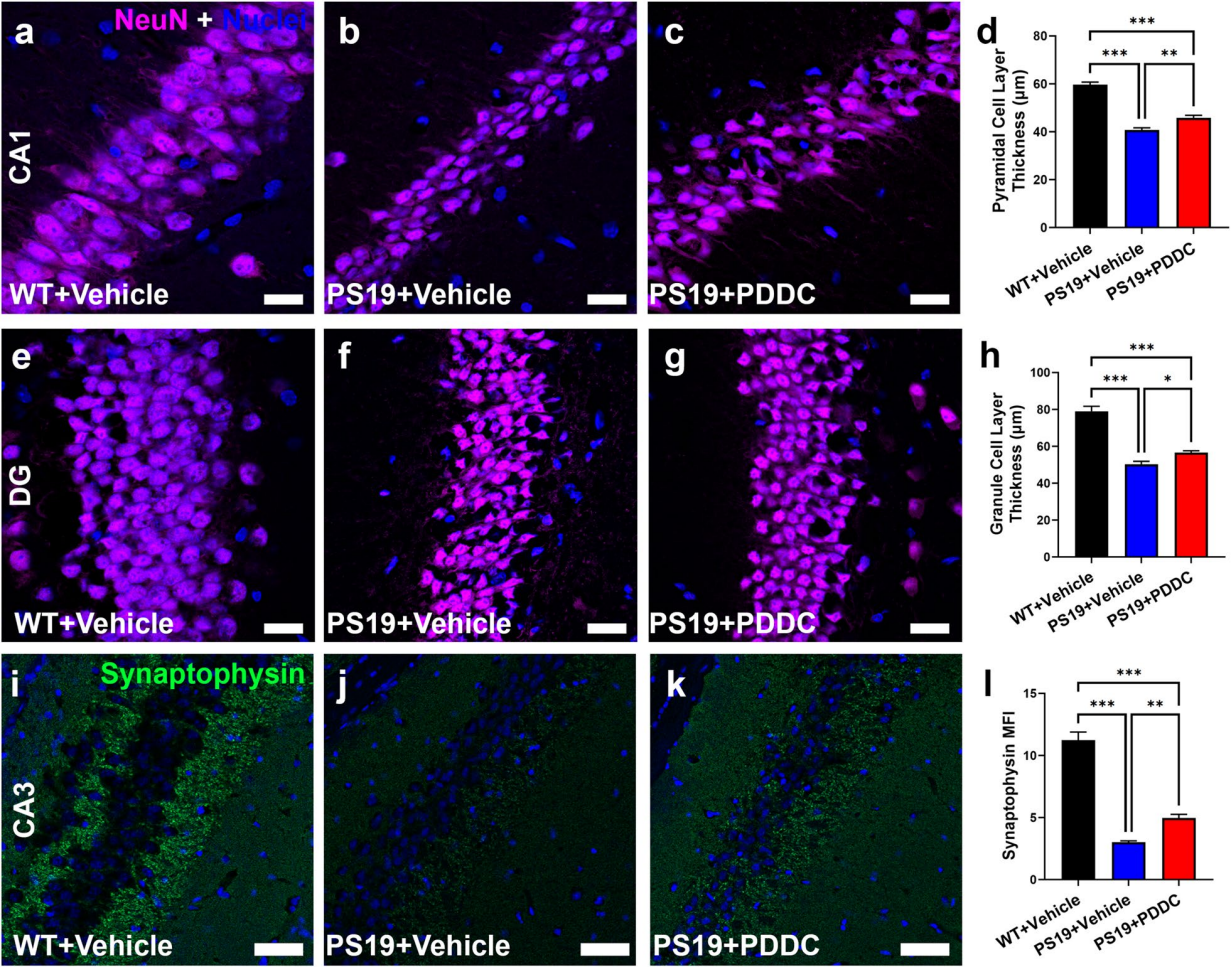
PDDC ameliorates hippocampal cell layer thinning and mossy fiber synaptophysin loss in PS19 mice. **a–h** Pyramidal cell layer thickness in the CA1 region (**a–d**) and granule cell layer thickness in the dentate gyrus (DG, **e–h**) of WT, vehicle-treated PS19 and PDDC-treated PS19 mice. Nuclei shown in blue. Scale bar, 20 µm. **d, h** Quantification of neuronal cell density counts from CA1 (**d**) and DG (**h**). *N* = 122–141 images from 7 to 8 mice/group. **i–l** Synaptophysin staining (green) of the mossy fiber layer in the CA3 from WT (**i**), vehicle-treated PS19 (**j**), and PDDC-treated PS19 (**k**) mice. Scale bars, 50 µm. **l** Quantification of the mean fluorescence intensity (MFI) of synaptophysin staining in the mossy fiber layer. Bars represent mean ± SEM. *P < 0.05, **P < 0.01, ***P < 0.001. *N* = 45–54 images from 5 to 6 mice/group. One-way ANOVA with Tukey’s multiple comparison

We observed thinning of the pyramidal cell layer in the CA1 region (Fig. 4a, b, d, *P* = 1.99 × 10^−11^) and the granule cell layer in the dentate gyrus (Fig. 4e, f, h, *P* = 1.79 × 10^−11^) in the PS19 mice compared to WT mice. PDDC treatment reduced this thinning (Fig. 4b–d, f–h, *P* = 0.00127 and *P* = 0.0118, respectively). Synaptophysin staining of mossy fibers in the CA3 region was reduced in the PS19 mice compared to the WT mice (Fig. 4i, j, l, *P* = 2.08 × 10^−12^). PDDC increased the synaptophysin staining compared to the vehicle-treated PS19 mice (Fig. 4j–l, *P* = 0.00923).

### PDDC treatment reduces glial activation in PS19 mice

**Fig. 5:**
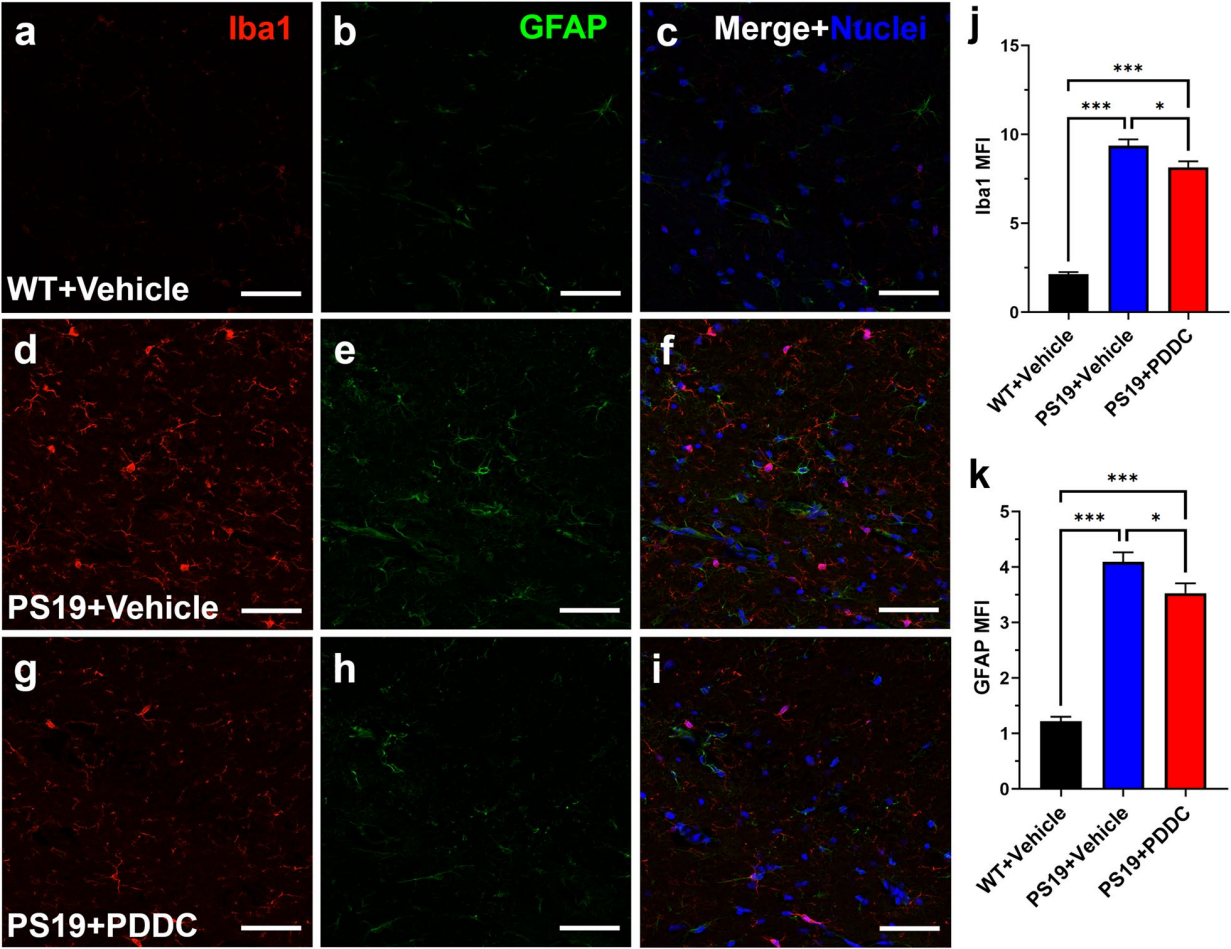
PDDC treatment reduces glial activation in the hippocampus of PS19 mice. Representative images from WT mice (**a–c**), vehicle-treated PS19 mice (**d–f**), and PDDC-treated PS19 mice (**g–i**). Microglia were stained with Iba1 (red). Astrocytes were stained with GFAP (green). Nuclei shown in blue. **j** Quantification of Iba1 MFI. **k** Quantification of GFAP MFI. *N* = 216–232 images from 9 to 10 mice/group. Bars represent mean ± SEM. **P < 0.01, ***P < 0.001. Scale bars, 50 µm. Gamma and brightness adjusted equally for all images. One-way ANOVA with Tukey’s multiple comparison

We previously reported that PDDC reduces EV release from astrocytes and activated microglia into plasma in a brain injury model [18, 20]. Given that glial overactivation has been observed in PS19 mice [34], we sought to evaluate the effect of PDDC treatment. The MFI of microglial Iba1 staining was elevated in PS19 mice compared to WT mice (Fig. 5a, d, j, *P* < 1.0 × 10^−15^). PDDC treatment in PS19 mice reduced Iba1 staining compared to the vehicle-treated PS19 mice (Fig. 5d, g, j, *P* = 0.0148). Similarly, astrocytic GFAP MFI was elevated in PS19 versus WT mice (Fig. 5b, e, k, *P* < 1.0 × 10^−15^), and PDDC treatment in the PS19 mice reduced the GFAP staining compared to the vehicle-treated PS19mice (Fig. 5e, h, k, *P* = 0.0298).

### PDDC treatment reduces the numbers of neuronal- and microglial-derived EVs and their tau content in the plasma of PS19 mice

**Fig. 6:**
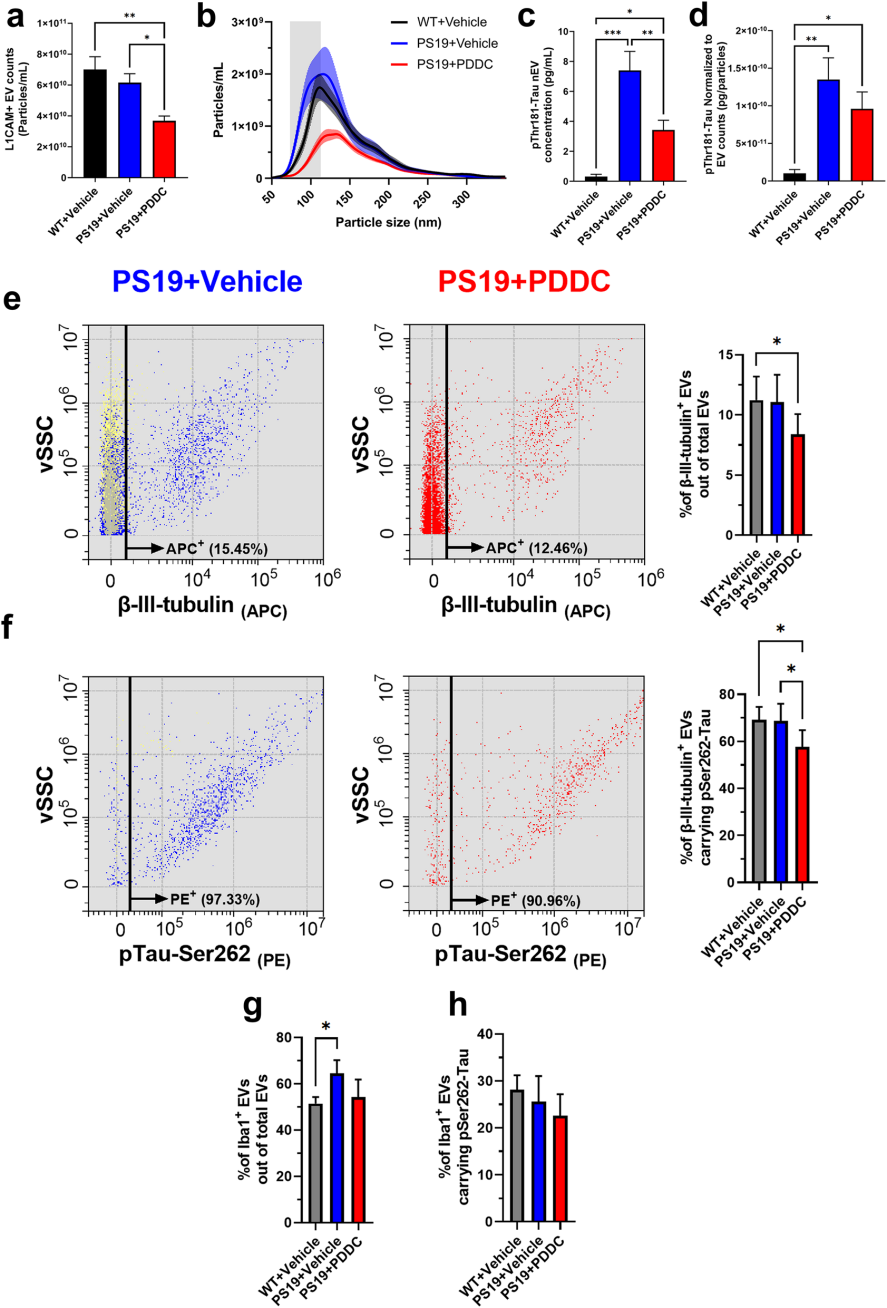
PDDC reduces plasma nEVs carrying pThr181-Tau in PS19 mice. **a** Quantification of L1CAM^+^ nEVs immunocaptured from the plasma of WT mice, vehicle-treated and PDDC-treated PS19 mice by NTA. *N* = 15–16. **b** Averaged size profiles of L1CAM^+^ nEVs from the plasma of WT mice, vehicle-treated and PDDC-treated PS19 mice. *N* = 15–16. **c** pThr181-Tau in lysed L1CAM^+^ nEVs from WT mice, vehicle-treated and PDDC-treated PS19 mice. *N* = 11–12. **d** pThr181-Tau normalized to nEV concentration from WT mice, vehicle-treated and PDDC-treated PS19 mice. *N* = 11–12. One-way ANOVA with Tukey’s multiple comparison. **e** Dot plots showing the vSSC vs APC-β-III-tubulin signal of BSE^+^ events gated in Fig. S5 for vehicle (left, blue events) and PDDC (middle, red events). Black line: threshold for APC-β-III-tubulin^+^ events. Yellow events indicate negative-control EVs labeled with BSE only. Bar graph: average percentage of APC-β-III-tubulin^+^ events out of total BSE^+^ events. **f** Dot plots showing the vSSC vs PE-pTau-Ser262 signal of APC-β-III-tubulin^+^ events gated in **b**. Black line: threshold for PE-pTau-Ser262^+^ signal. Bar graph: average percentage of APC-β-III-tubulin^+^ events double-positive for PE-pTau-Ser262. **g, h** Mean percentage of APC-Iba-1^+^ events out of total BSE^+^ events (**g**) or APC-Iba-1^+^ events double-positive for PE-pTau-Ser262 (**h**) for each group. **e–h** Two-way ANOVA. Bars represent mean ± SEM. *P < 0.05, **P < 0.01, ***P < 0.001

Studies leveraging plasma samples from large longitudinal aging studies have found that the pTau cargo in nEVs is a prognostic indicator of cognitive decline and AD diagnosis [51, 52]. Therefore, we sought to evaluate the effects of PDDC on the number, size, and tau content of nEVs isolated from the plasma of PS19 mice. Flow cytometry analysis at the single-EV level and ExoView confirmed that L1CAM immunocapture resulted in the recovery of detergent-sensitive membranous nanoparticles carrying canonical transmembrane and intravesicular EV markers (Additional file 1: Fig. S5). NTA revealed that the PDDC-treated PS19 mice had reduced plasma nEV concentration when compared to both WT and vehicle-treated PS19 mice (Fig. 6a, *P* = 0.001 and *P* = 0.0187, respectively), mainly driven by a decrease of small EVs (< 150 nm diameter) (Fig. 6b). We found higher levels of pThr181-Tau in lysed nEVs isolated from vehicle-treated PS19 mice compared to WT controls (Fig. 6c, *P* = 5.57 × 10^−6^), whereas PDDC treatment in PS19 mice reduced the nEV pThr181-Tau (*P* = 0.0061). After normalization to L1CAM^+^ EV concentration, pThr181-Tau showed significant increases in both vehicle- and PDDC-treated PS19 mice compared to WT (*P* = 0.0016 and *P* = 0.033, respectively), with a trend of reduction with PDDC treatment compared to vehicle in PS19 mice (Fig. 6d).

At the single-EV level via flow cytometry analysis, the concentration of total plasma EVs identified using the fluorescent EV marker BSE remained unchanged in all treatment groups (Additional file 1: Fig. S6). PDDC-treated PS19 mice had decreased nEV percentages, identified using the neuron-specific marker β-III-tubulin, compared to both WT and vehicle-treated PS19 mice (Fig. 6e, *P* = 0.041), consistent with NTA findings for immunocaptured L1CAM^+^ nEVs. PDDC treatment also reduced β-III-tubulin^+^ nEVs carrying pSer262-Tau compared to vehicle-treated WT and PS19 mice (Fig. 6f, *P* = 0.022 and *P* = 0.027, respectively). Flow cytometry analysis was also used to assess the effect of PDDC on EVs expressing Iba-1, a subpopulation enriched in microglia-derived EVs (mEVs). The vehicle-treated PS19 mice exhibited a higher percentage of Iba-1^+^ EVs compared to WT controls (*P* = 0.025), and PDDC treatment induced a trend of decrease of the percentage (Fig. 6g). PDDC also mediated a trend of reduction of Iba-1^+^ EVs carrying pSer262-Tau (Fig. 6h).

### PDDC treatment reduces tau spread in an AAV mutant hTau propagation model

**Fig. 7:**
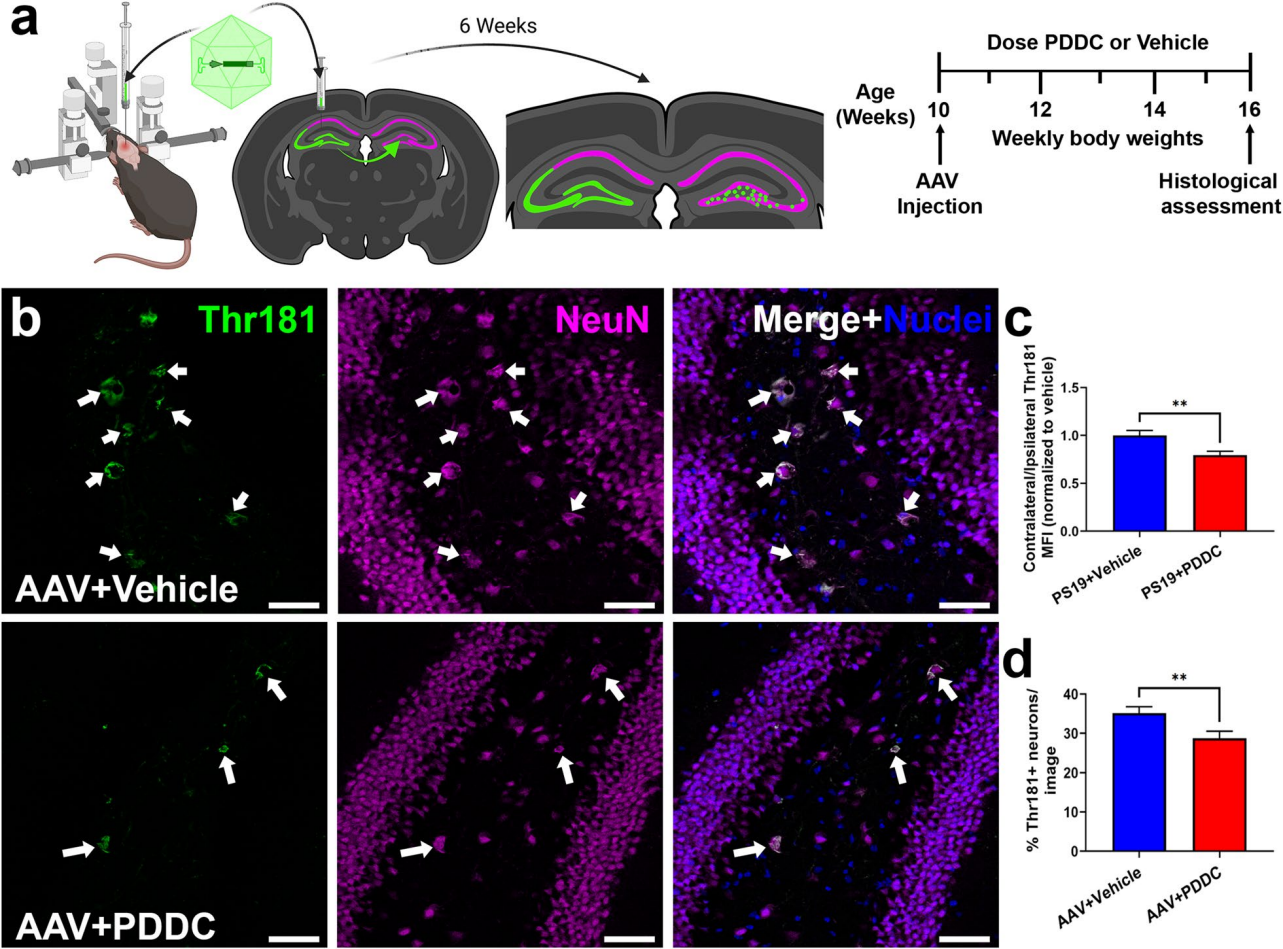
PDDC treatment reduces tau spread in an AAV mutant hTau propagation model. **a** AAV-hTau model and dosing schematic. Mice were stereotaxically injected at 10 weeks old into the left dorsal hippocampus with AAV-CBA-hTau24(P301L)(S320F)-WPRE which was taken up and expressed by cells in the left CA3 and dentate gyrus and propagated to the right DG hilus region over the course of 6 weeks. **b** Representative images of the contralateral DG showing pThr181-Tau staining (green) from vehicle-treated (top) and PDDC-treated (bottom) AAV-hTau mice. Neurons stained with NeuN (magenta). Nuclei shown in blue. Scale bars, 50 µm. Gamma and brightness adjusted equally for all images presented. **c** Quantification of pThr181-Tau MFI of the contralateral DG normalized to the ipsilateral DG pThr181-Tau MFI. *N* = 81–84 images/group from 17 mice/group. **d** Quantification of the percentage of pThr181-Tau^+^ neurons in the contralateral dentate gyrus. *N* = 56–72 images/group from 17 mice/group. Unpaired two-tailed *t*-test. **P < 0.01. Bars represent mean ± SEM

Building upon similar models [16, 33, 53], our group developed a rapid tau propagation model where an AAV vector expressing double mutant hTau (P301L/S320F) was unilaterally injected into the hippocampus and the propagation of the hTau to the contralateral hippocampus was monitored over 6 weeks [15], during which time the animals were treated with either vehicle or PDDC-containing chow (Fig. 7a). The PDDC-treated PS19 mice exhibited a significant reduction of contralateral pThr181-Tau MFI compared to the vehicle-treated PS19 mice (Fig. 7b, c, *P* = 0.0052). There were also significantly fewer NeuN-positive neurons expressing pThr181-Tau in the contralateral hippocampus after PDDC treatment compared with vehicle treatment (Fig. 7b, d, *P* = 0.0096). Ceramides were also altered with PDDC treatment. Of the 46 ceramides detected, 4 were significantly reduced in the hippocampus of PDDC-treated mice compared to vehicle (Additional file 1: Fig. S7 and Table S5). The ceramide levels with PDDC treatment were similar to the unaffected cortical levels, consistent with our prior study showing that nSMase2 activity levels are selectively elevated in the tau-expressing hippocampus but not in the tau-naïve cortex [15].

## Discussion

**Fig. 8:**
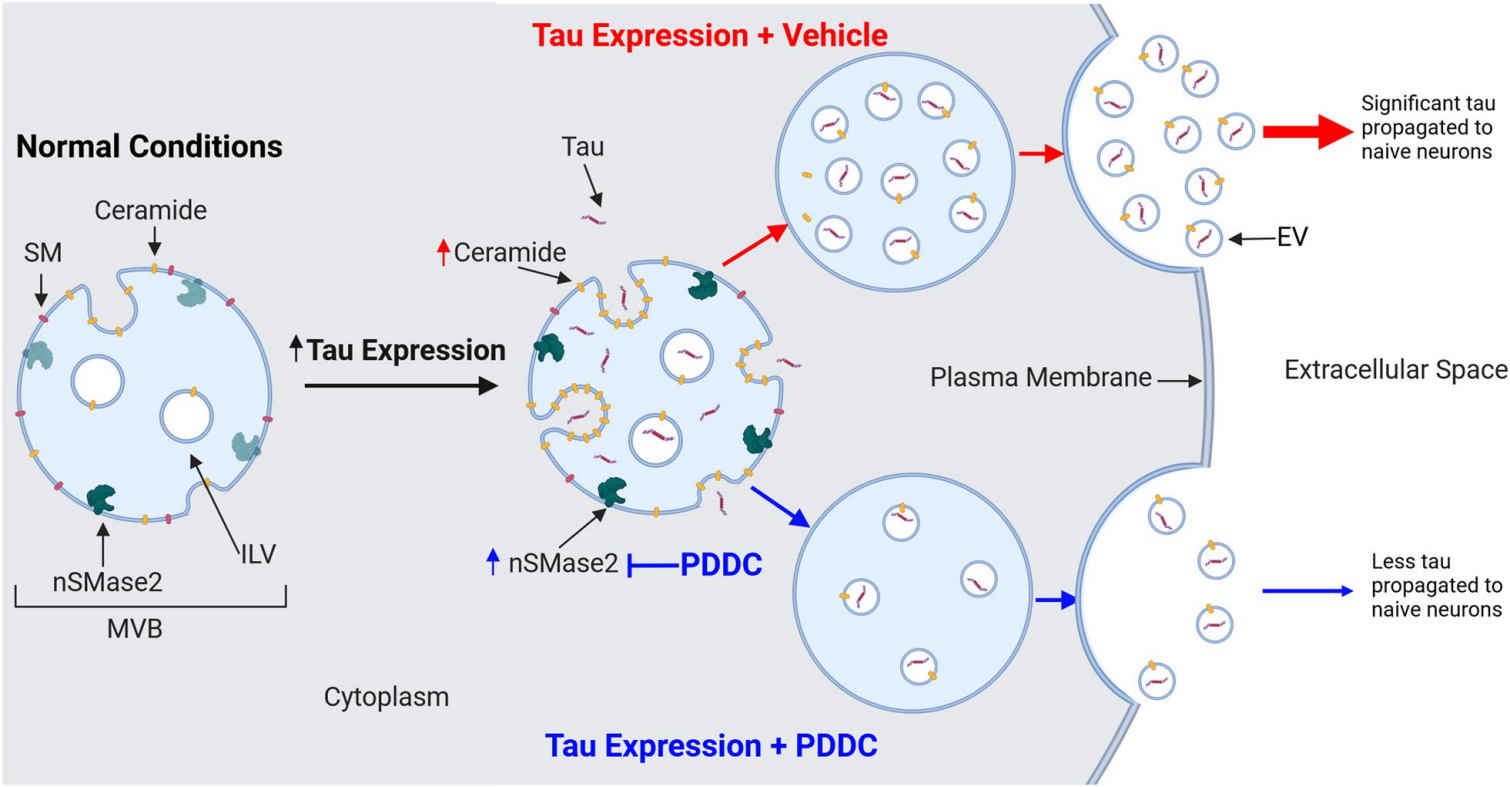
Summary of nSMase2’s role in EV-mediated tau propagation. Under normal MVB conditions, nSMase2 is active at basal levels with moderate ceramide levels. With tau expression, nSMase2 activity is increased and ceramide levels rise, leading to the invagination of the MVB membrane, forming more ILVs and trapping tau within, which are then released to the extracellular space as EVs carrying tau (black, upper pathway). These EVs can then pass on tau to naïve, healthy neurons, thus propagating the disease. With PDDC (blue, lower pathway), nSMase2 is inhibited and the amount of ceramides is reduced, forming fewer ILVs and trapping less tau which causes fewer naïve, healthy neurons to become seeded

In this study, we demonstrate that the potent and selective nSMase2 inhibitor PDDC chronically administered to PS19 mice is well-tolerated and can achieve sustained levels in the brain at concentrations capable of nSMase2 inhibition. Like in human AD patients [21–26], we show, both in vitro and in vivo, that neurons expressing mutant human tau exhibit elevated ceramide levels. In both the PS19 and the AAV-hTau-transduced mice, we found elevated brain nSMase2 enzymatic activity and ceramide levels in the hippocampus with tau expression. Both of these elevations were normalized by PDDC treatment, confirming brain target engagement. Also similar to human AD [27–29], the PS19 mice exhibited elevated brain tau, thinning of the hippocampal neuronal cell layers, decreased mossy fiber synaptophysin staining, and elevated glial activation, all of which were improved with PDDC. Additionally, the PS19 mice exhibited increased tau content in their circulating plasma nEVs, which was reduced with PDDC treatment. This is translationally exciting as studies leveraging plasma samples from large longitudinal studies of aging have shown that the pTau cargo in nEVs can predict future AD diagnosis [54] and the development of cognitive decline in at-risk populations [55]. And lastly, using a newly developed AAV-hTau injection propagation model, we directly demonstrated that PDDC treatment inhibited the spread of mutant hTau between synaptically connected brain regions. A summary of our findings is detailed in Fig. 8.

In addition to their role in EV biogenesis [56], ceramides are elevated in AD patient brain tissues [21, 25, 26], plasma [23, 24], and CSF [22] and have been hypothesized to dysregulate autophagy, impair mitochondrial health, and induce senescence (reviewed in [57]). Here we show, for the first time, that several long-chain and very-long-chain ceramides are significantly elevated in cultured neurons expressing mutant human tau, in the PS19 mouse brain, and specifically in the tau-expressing hippocampus of AAV-hTau mice. Treatment with PDDC reduced all aberrant ceramide species and in most cases, completely normalized ceramides to WT baseline levels. In particular, ceramides d18:1/16:0, d18:1/18:0, and d18:1/18:1 which were increased with tau expression, were reduced by PDDC treatment. The elevations of long-chain ceramides have also been reported in human AD. For example, elevation of d18:1/18:0 has been reported in human AD brains and investigations into the oxidative stress induced by Aβ suggest that the dysregulated ceramide production enhances neuronal dysfunction and death [58]. Furthermore, in brain tissues from frontotemporal dementia Pick’s disease patients, the d18:1/16:0 ceramide is elevated in association with astrocytes and hypothesized to be proinflammatory [59]. Thus, reducing these long-chain ceramides with PDDC may produce beneficial effects by reducing neuroinflammation observed in AD patients. In support of this, we observed lower Iba1 and GFAP staining intensities in mice treated with PDDC.

Ceramides are critical for EV biogenesis, as their enrichment at the membrane enhances curvature and induces eventual EV budding both at the plasma membrane and within multi-vesicular bodies [12]. EVs are thought to play a role in the pathological spread of tau throughout the AD brain in a predictable manner along connectivity pathways in a prion-like fashion [3]. Mounting evidence has correlated tau propagation with disease progression [60, 61]. The data from both the PS19 transgenic and the AAV-tau propagation models show that PDDC reduces tau spread by decreasing the absolute number of neurons seeded with pathological tau. We observed that PDDC treatment induced a significant reduction in the number of tau-positive neurons in the contralateral DG in the mice injected with AAV-hTau, compared with vehicle treatment. In the PS19 mice, while most hippocampal neurons had some tau expression, the intensity of the tau fluorescence measured individually within the neurons was significantly reduced by PDDC treatment. These data suggest that PDDC did not alter the basal rate of tau accumulation driven by the neuronal promoter, but was inhibiting an alternative mechanism responsible for additional accumulation of tau in neurons, which may be driven by EVs. Together, these data suggest that nSMase2 inhibition reduces hippocampal tau burden by slowing tau propagation through reduced production of tau-containing EVs.

This hypothesis is further supported by our assessment of nEVs isolated from the plasma of PS19 mice. Biomarker studies in humans have shown that pThr181-Tau and pThr231-Tau in plasma nEVs predict AD diagnosis [52]. Similar as in AD patients, we found that pThr181-Tau levels in plasma nEVs were increased in the PS19 mice, while chronic PDDC treatment reduced this increase. In addition, PDDC decreased the number of nEVs in plasma, especially smaller-sized nEVs. Somewhat surprisingly, we did not observe a PDDC-induced reduction in total EVs in plasma. It is possible that the nEV biogenesis (or at least the L1CAM^+^ EV biogenesis) is predominantly regulated via the nSMase2 pathway over other pathways, such as ESCRT-mediated pathways. As a consequence, the L1CAM-based isolation could have biased towards recovering EVs generated via the nSMase2 pathway and as such the L1CAM^+^ EV population demonstrated a reduction with PDDC, while the total EV population did not. In addition, this study focused on the levels of ceramides affected by PDDC treatment; however, future studies examining whether there are alterations of ESCRT proteins with PDDC treatment would be valuable and could shed light on possible compensatory mechanisms.

With regards to the EV content, using flow cytometry analysis, we observed at the single EV level a significant reduction in the percentage of nEVs containing pSer262-Tau. Taken together, these data demonstrate that PDDC treatment results in reduced circulating nEVs and, within this population, a lower percentage of pTau-carrying nEVs, thus reducing the overall tau seeding potential. This is therapeutically important as recent evidence suggests that seeding is likely a rate-limiting step in AD disease progression [62]. Reducing the number of tau-seeds via inhibiting the formation and/or spread of tau-carrying EVs represents a novel and to date unexplored therapeutic avenue for AD. While reducing the nEV population has therapeutic benefits, there may also be unintended negative effects given the importance of EVs for cell-to-cell communication. As such, further studies are needed to elucidate whether there are toxicities related to blockade of nEV generation. However, it is noteworthy that even with a 100 mg/kg PDDC dose which provides nSMase2-inhibitory exposures in the brain continually over 24 h [20], nEVs were not completely inhibited and no adverse effects were observed.

The therapeutic benefit of nSMase2 inhibition may be further amplified in AD patients given that Aβ, the other pathological protein of AD, has also been observed to be associated with peripheral nEVs [63, 64]. Like tau, Aβ also has seeding potential [65]. Therefore, by reducing Aβ packaged into EVs, the negative effects of spreading Aβ pathology may also be mitigated. This possible benefit is supported by the recent positive results with the clearance of Aβ via targeted antibody therapeutics demonstrating that reducing the Aβ burden is beneficial in AD patients [1]. Additionally, we have previously observed positive results with nSMase2 inhibition in the 5 × FAD AD mouse model, which only exhibits Aβ pathology [19].

In addition to tau accumulation, the PS19 mice showed synaptic loss and thinning of hippocampal cellular layers [34, 42, 66, 67]. Importantly, these pathological features have also been observed in human AD [27–29]. PDDC improved the cell layer thickness of both the CA1 and dentate gyrus regions as well as increasing the synaptophysin staining in the mossy fiber layer of the CA3 region in PS19 mice. Since tau aggregation correlates with AD disease progression [60, 61] and studies utilizing human iPSC-derived neurons show that tangle formation precedes neuronal death [68], reducing tau propagation via PDDC may meaningfully attenuate tau aggregation and delay neurotoxicity, contributing to improved cell layer thickness and maintenance of mossy fibers. These results suggest that by decreasing the spread of tau, PDDC also affords neuroprotective benefits.

Microglia and astrocytes have also been implicated in neuronal cell loss observed in AD (see review [69]). Because both astrocyte- [70] and microglia-derived [16] EVs carry tau, and cortical glial reactivity positively correlates with AD neurofibrillary tangle burden [71], it is reasonable to suggest that at least some of the glial-mediated pathological contributions in AD stem from their role in the spread of tau. In fact, previous studies in both a rapid tau propagation model and the PS19 model showed that microglial depletion reduces tau burden [16]. We therefore hypothesized that PDDC’s therapeutic benefit could, in part, be due to its impact on glia. Here we report that PDDC inhibited glial activation in PS19 mice. Specifically, we observed reduced intensity of both Iba1 and GFAP hippocampal staining. Moreover, we observed trends for PDDC reducing the percentage of plasma Iba-1^+^ EVs and the percentage of Iba-1^+^ EVs that are double-positive for PE-pTau-Ser262. These findings are in line with prior studies in an acute brain injury model showing that PDDC reduces the release of EVs into the systemic circulation from CD11b^+^ activated microglia [20].

While the results of this study are exciting, they have limitations. It has been hypothesized that the specificity of L1CAM immunocapture towards blood-borne nEVs is suboptimal based on evidence of L1CAM expression by potential peripheral sources of circulating L1CAM^+^ EVs, and L1CAM secretion via proteolytic membrane shedding [72–75]. These alternative mechanisms of L1CAM release could supply the blood with non-neuronal EVs and free L1CAM peptides, which interfere with the recovery of nEVs via L1CAM immunocapture, limiting the potential of this methodology to provide biomarkers of neuronal function in blood. Yet, to this date, experiments have not directly addressed these hypotheses. On the other hand, cumulative evidence supports the use of L1CAM as a target for the isolation of blood-borne nEVs, including the enrichment of EV and neuronal markers in L1CAM IP eluates from human blood as well as the correlation of EV cargo with brain disease states and therapeutic interventions [54, 76–84], the recovery of GFP-positive EVs from the plasma of Nestin-GFP mice selectively expressing GFP in neurons [79], and the positive correlations between brain and nEV levels of pathological proteins in multiple AD mouse models [43]. In addition, proteomic analyses have detected L1CAM in EVs isolated from human brains [85, 86] and that a high percentage of proteins carried by EVs isolated from human blood via L1CAM IP are highly expressed in the human brain and shared by EVs isolated from the conditioned media of human neurons in culture [83, 87]. An additional limitation is that the histological methods employed for tau, NeuN and synaptophysin do not provide the most sensitive method  for assessing the subtle changes of cell content as well as microglial and astrocytic activation states. Future studies examining these changes with flow cytometry analysis utilizing specific markers for astrocytic and microglial activation, would provide more in-depth insights into how the individual cells are responding to PDDC treatment. The improvement of the cell layer thickness and synaptophysin staining with PDDC treatment also would benefit from further exploration, particularly with robust behavioral characterization and long-term potentiation recording experiments.

## Conclusions

The present study demonstrates that PDDC has multifaceted effects on the pathology of AD. This potent and selective nSMase2 inhibitor not only directly reduced tau propagation, but also decreased hippocampal gliosis as well as neuronal and synaptic degeneration, and normalized brain ceramide levels. From a therapeutic perspective, this is exciting as these abnormalities have all been described in human AD. PDDC also reduced the number of nEVs and absolute pTau levels in nEVs found in PS19 mouse plasma. Again, from a therapeutic perspective, this is important as increased tau in nEVs of AD patients has been shown to positively correlate with disease progression. While future toxicological studies are needed to thoroughly assess the tolerability of chronic PDDC prior to clinical trials, based on the current data, PDDC represents a promising new therapeutic with the potential to slow the progression of AD by reducing the spread of hyperphosphorylated tau species.

### Supplementary Information

**Additional file 1: Supplementary Methods. Table S1** Ceramide values and  *P* values from neuronal cultures. **Table S2** Modified SHIRPA assessment did not reveal abnormalities with PDDC treatment. **Table S3** Clinical chemistry values are within normal range. **Table S4** Ceramide values and  *P* values from PS19 mice. **Table S5** Ceramide values and  *P* values from AAV-hTau mice. **Figure S1** AAV-GFP and AAV-hTau infected cells express appropriate proteins. **Figure S2** PDDC treatment does not negatively affect behavior in WT or PS19 mice. **Figure S3** PDDC does  not alter Sarkosyl-soluble and -insoluble tau fractions. **Figure S4** Uncropped western blot images. **Figure S5** Characterization of L1CAM^+^ nEVs immunoprecipitated from mouse plasma. **Figure S6** PDDC does not affect total EVs by FCA analysis. **Figure S7** PDDC reduces ceramide levels in the hippocampus of AAV-hTau mice.

## Data Availability

All data presented in this study are available within the manuscript and supplementary material.

## Abbreviations

AD: Alzheimer’s disease
Aβ: Amyloid-beta
pTau: Hyperphosphorylated tau
EV: Extracellular vesicle
nSMase2: Neutral sphingomyelinase 2
PDDC: Phenyl(R)-(1-(3-(3,4-dimethoxyphenyl)-2,6-dimethylimidazo[1,2-b]pyridazin-8-yl)pyrrolidin-3-yl)-carbamate
AAV: Adeno-associated virus
PFA: Paraformaldehyde
SM: Sphingomyelin
nEV: Neuronal EV
NTA: Nanoparticle tracking analysis
MFI: Mean fluorescence intensity
