# Supplementary material for: Inhibiting tau-induced elevated nSMase2 activity and ceramides is therapeutic in an Alzheimer’s disease mouse model
Source: Transl Neurodegener. 2023 Dec 4;12:56. doi: 10.1186/s40035-023-00383-9 (PMC10694940; doi:10.1186/s40035-023-00383-9)
Supplement: Supplementary file 1 — Additional file 1: Supplementary Methods. Table S1 Ceramide values and P values from neuronal cultures. Table S2 Modified SHIRPA assessment did not reveal abnormalities with PDDC treatment. Table S3 Clinical chemistry values are within normal range. Table S4 Ceramide values and P values from PS19 mice. Table S5 Ceramide values and P values from AAV-hTau mice. Figure S1 AAV-GFP and AAV-hTau infected cells express appropriate proteins. Figure S2 PDDC treatment does not negatively affect behavior in WT or PS19 mice. Figure S3 PDDC does not alter Sarkosyl-soluble and -insoluble tau fractions. Figure S4 Uncropped western blot images. Figure S5 Characterization of L1CAM+ nEVs immunoprecipitated from mouse plasma. Figure S6 PDDC does not affect total EVs by FCA analysis. Figure S7 PDDC reduces ceramide levels in the hippocampus of AAV-hTau mice. [file 40035_2023_383_MOESM1_ESM.docx]

**SUPPLEMENTARY METHODS**

**Open field testing**

Mice were placed in an open field apparatus (Photobeam Activity System, San Diego Instruments, San Diego, CA) and allowed to move freely over a 30 min testing period. Photobeam breaks were recorded in 3 min bins and the sum of the entire 30 min was used for analysis.

**Modified SHIRPA**

After 5 months on treatment and prior to sacrifice, animals underwent a modified SHIRPA assessment as previously described(*1, 2*). Briefly, a blinded experimenter examined the animals individually and scored them on 20 characteristics based on the original SHIRPA protocol. Comparisons were made using Kruskal-Wallis test with Dunn’s test for multiple comparisons.

**Clinical Chemistry**

Blood was collected via cardiac puncture into uncoated tubes at the time of sacrifice following 5 months of treatment. Blood was allowed to coagulate for 30 min at room temperature before being spun down at 1500 x *g* for 10 mins and the serum was collected and frozen prior to storage at -80°C. Serum was shipped on dry ice to IDEXX BioAnalytics (North Grafton, MA, USA) for analysis in their comprehensive chemistry panel.

**Table S1. Ceramide values and *P* values from neuronal cultures.**

|  | **Control** | | **AAV-GFP** | | | **AAV-hTau** | | | |
| --- | --- | --- | --- | --- | --- | --- | --- | --- | --- |
| **Ceramides** | **pg/mg** | **SD** | **pg/mg** | **SD** | ***P* value vs Control** | **pg/mg** | **SD** | ***P* value vs Control** | ***P* value vs AAV-GFP** |
| **Cer d18:1/18:0** | 389.3 | 62.55 | 415.8 | 54.65 | 0.7739627 | 603.5 | 43.09 | ***0.0008663*** | ***0.0008663*** |
| **Cer d18:1/16:0** | 234.5 | 70.45 | 236 | 24.78 | 0.99892496 | 398.1 | 28.66 | ***0.0018885*** | ***0.0020045*** |
| **Cer d18:1/24:0** | 171.7 | 28.4 | 178.3 | 19.64 | 0.90911796 | 246.4 | 17.53 | ***0.0027671*** | ***0.0049929*** |
| **HexCer d18:1/18:1** | 10.66 | 1.792 | 11.3 | 0.2922 | 0.69212032 | 14.27 | 0.4617 | ***0.0028173*** | ***0.0093726*** |
| **HexCer d18:1/24:1** | 14.49 | 1.984 | 15.54 | 0.9137 | 0.6365753 | 18.42 | 1.696 | ***0.017125*** | **0.07244547** |
| **Cer d18:1/18:1** | 9.982 | 1.9 | 11.81 | 2.603 | 0.49286426 | 14.68 | 2.014 | ***0.0347286*** | **0.20996014** |
| **DHCer d18:0/24:0** | 3.494 | 0.7432 | 3.373 | 0.4729 | 0.9860488 | 5.563 | 1.64 | **0.05578147** | ***0.0434789*** |
| **HexCer d18:1/18:0** | 7.575 | 1.651 | 6.897 | 0.9392 | 0.71950752 | 5.575 | 0.9168 | 0.10332966 | 0.32066341 |
| **Cer d18:1/22:0** | 69.74 | 12.99 | 56.65 | 21.03 | 0.48863471 | 49.78 | 10.78 | 0.21974343 | 0.81066964 |
| **DHCer d18:0/22:0** | 6.285 | 0.8757 | 6.26 | 0.8064 | 0.9994154 | 7.628 | 1.447 | 0.23828249 | 0.22766432 |
| **Cer d18:1/24:1** | 3.176 | 0.5081 | 3.267 | 0.5479 | 0.96576268 | 3.712 | 0.4631 | 0.33940368 | 0.46113096 |
| **HexCer d18:1/24:0** | 2.959 | 0.4548 | 2.839 | 0.6478 | 0.93999355 | 2.437 | 0.3714 | 0.3523482 | 0.52299501 |
| **HexCer d18:1/16:1** | 0.2676 | 0.02163 | 0.2681 | 0.01316 | 0.99949577 | 0.2499 | 0.02814 | 0.51054853 | 0.49391663 |
| **Hex2Cer d18:1/24:1** | 1.092 | 0.2708 | 0.9449 | 0.2407 | 0.71329224 | 0.9 | 0.2703 | 0.57101155 | 0.96801778 |
| **DHHexCer d18:0/18:0** | 2.395 | 0.6924 | 2.339 | 0.378 | 0.98873105 | 2.067 | 0.5284 | 0.68613269 | 0.76867666 |
| **Cer d18:1/20:0** | 173.6 | 42.81 | 163.2 | 28.44 | 0.89516285 | 159.5 | 23.21 | 0.81699424 | 0.98573805 |
| **HexCer d18:1/20:1** | 22.79 | 3.063 | 30.95 | 14.78 | 0.46637153 | 26.35 | 6.018 | 0.85591228 | 0.77292235 |
| **Hex2Cer d18:1/18:1** | 0.5566 | 0.1756 | 0.6684 | 0.2178 | 0.69250472 | 0.495 | 0.1719 | 0.89160233 | 0.43338442 |
| **Cer d18:1/20:1** | 21.4 | 6.218 | 27.18 | 6.729 | 0.40044116 | 22.93 | 4.956 | 0.93174914 | 0.59525197 |
| **HexCer d18:1/22:1** | 7.823 | 0.9081 | 7.994 | 0.9437 | 0.95019744 | 7.673 | 0.3912 | 0.96064308 | 0.83608067 |
| **Cer d18:1/16:1** | 0.1406 | 0.03848 | 0.1412 | 0.06056 | 0.99989991 | 0.1401 | 0.073 | 0.99991392 | 0.99962826 |
| **DHHexCer d18:0/24:0** | 8.748 | 0.9889 | 8.769 | 0.8852 | 0.99937997 | 8.743 | 0.8057 | 0.99996342 | 0.99904247 |

Concentrations of the 22 detected ceramides in uninfected, AAV-GFP infected, and AAV-hTau infected primary hippocampal cells. SD=Standard Deviation. Red indicate statistically significant ceramides.

**Table S2. Modified SHIRPA assessment did not reveal abnormalities with PDDC treatment.**

| **Parameter** | **WT+Vehicle** | **WT+PDDC** | **PS19+Vehicle** | **PS19+PDDC** |
| --- | --- | --- | --- | --- |
| **Fur Color** | 2 (2-2) | 2 (2-2) | 2 (2-2) | 2 (2-2) |
| **Hair Length** | 0 (0-0) | 0 (0.25-0) | 0 (0-0) | 0 (0-0) |
| **Respiration** | 2 (2-2) | 2 (2-2) | 2 (2-2) | 2 (2-2) |
| **Tremor** | 0 (0-0) | 0 (0.25-0) | 0 (0-0) | 0 (0-0) |
| **Body Position** | 3 (3-3) | 3 (4-3) | 3 (3-3) | 3 (3-3) |
| **Transfer Arousal** | 5 (5-5) | 5 (5-4.75) | 5 (5-5) | 5 (5-4) |
| **Startle Response** | 1 (1-0) | 2 (2.25-1) | 1 (2-0.5) | 1 (2-1.5) |
| **Gait** | 0 (0-0) | 0 (0-0) | 0 (0-0) | 0 (0-0) |
| **Touch Escape** | 1 (2-1) | 2 (3-2) | 2 (2-1) | 2 (3-1) |
| **Trunk Curl** | 0 (0-0) | 0 (0-0) | 0 (0-0) | 0 (0-0) |
| **Grip Strength** | 2.5 (3-2) | 3 (3-3) | 3 (3-3) | 3 (3-2.5) |
| **Pinna Reflex** | 1 (1-1) | 1 (1-1) | 1 (1-1) | 1 (1-1) |
| **Toe Pinch** | 3 (3-3) | 3 (3-3) | 3 (3-3) | 3 (3-3) |
| **Corneal Reflex** | 0 (0-0) | 0 (0-0) | 0 (0-0) | 0 (0-0) |
| **Whisker Appearance** | 0 (0.75-0) | 1 (1-0) | 0 (0.5-0) | 0 (1-0) |
| **Bite Reflex** | 0 (0-0) | 0 (0-0) | 0 (0-0) | 0 (0-0) |
| **Righting Reflex** | 0 (0-0) | 0 (0-0) | 0 (0-0) | 0 (0-0) |
| **Fear/Freezing** | 0 (0-0) | 0 (0.25-0) | 0 (0-0) | 0 (0-0) |
| **Aggression** | 0 (0-0) | 0 (0-0) | 0 (0-0) | 0 (0-0) |
| **Vocalization** | 0 (0-0) | 0 (0-0) | 0 (0.5-0) | 0 (0-0) |

Values from WT and PS19 mice treated with vehicle or PDDC chow for 5 months. Values given represent mean. Values in parenthesis give upper and lower quartiles. N=6-12/group.

**Table S3. Clinical chemistry values are within normal range.**

| **Parameter** | **WT+Vehicle** | **WT+PDDC** | **PS19+ Vehicle** | **PS19+ PDDC** |
| --- | --- | --- | --- | --- |
| **ALB (g/dL)** | 3.125±0.111 | 2.975±0.103 | 3.167±0.0577 | 3.133±0.0577 |
| **ALP (U/L)** | 101±15.416 | 181.5±28.869 | 72.67±21.733 | 62.33±8.021 |
| **ALT (U/L)** | 55.5±14.315 | 52.25±15.052 | 21.33±7.572 | 18.67±1.528 |
| **AST (U/L)** | 177.8±37.435 | 137±25.093 | 80.67±18.556 | 55.67±16.073 |
| **BUN (mg/dL)** | 24.25±7.284 | 24.25±6.663 | 18.67±5.508 | 18.67±1.155 |
| **BUN/CREAT** | 79.17±35.365 | 30.05±9.484 | 120±42.72 | 82.22±13.472 |
| **Ca (mg/dL)** | 10.45±0.477 | 9.725±0.0479 | 10.5±0.265 | 10.33±0.208 |
| **CK (U/L)** | 309.5±85.842 | 76.75±39.112 | 245±134.1 | 134.667±73.935 |
| **CREAT (mg/dL)** | 0.45±0.119 | 0.8333±0.0333 | 0.1667±0.0577 | 0.233±0.0577 |
| **GLUC (mg/dL)** | 191.8±25.214 | 206.3±16.889 | 216.67±10.214 | 220±39.154 |
| **LDH (U/L)** | 404.3±72.554 | 260±15.529 | 225±60.556 | 173.67±63.003 |
| **PHOS (mg/dL)** | 9.4±0.803 | 8.6±0.998 | 6.867±0.115 | 7.367±0.586 |
| **TBILI (mg/dL)** | 0.1±0 | 0.475±0.144 | 0.2667±0.0577 | 0.233±0.577 |
| **TP (g/dL)** | 5.725±0.390 | 5.2±0.108 | 5.633±0.153 | 5.6±0.173 |

Clinical chemistry values for WT and PS19 mice treated with either vehicle or PDDC chow. ALB = albumin; ALP = alkaline phosphatase; ALT = alanine aminotransferase; AST = aspartate aminotransferase; BUN = blood urea nitrogen; Ca = calcium; CK = creatinine kinase; CREAT = creatinine; GLUC = glucose; LDH = lactate dehydrogenase; PHOS = phosphorus; TBILI = total bilirubin; TP = total protein. Values given represent mean ± SD. N=3/group

**Table S4. Ceramide values and *P* values from PS19 mice.**

|  | **WT + Vehicle** | | **WT + PDDC** | | | **PS19 + Vehicle** | | | | **PS19 + PDDC** | | | | |
| --- | --- | --- | --- | --- | --- | --- | --- | --- | --- | --- | --- | --- | --- | --- |
| **Ceramides** | **ng/mg** | **SD** | **ng/mg** | **SD** | ***P* value vs WT+Veh** | **ng/mg** | **SD** | ***P* value vs WT+Veh** | ***P* value vs WT+PDDC** | **ng/mg** | **SD** | ***P* value vs WT+Veh** | ***P* value vs WT+PDDC** | ***P* value vs PS19+Veh** |
| ***Cer d18:1/18:1*** | 15.14 | 2.749 | 11.12 | 1.785 | 0.501985 | 34.66 | 9.351 | ***5.62E-08*** | *3.145E-08* | 15.44 | 4.777 | 0.999367 | 0.4721552 | ***2.026E-07*** |
| ***Cer d18:1/16:0*** | 344.9 | 49.92 | 226.1 | 29.67 | 0.120266 | 768.4 | 162.8 | ***1.17E-09*** | *1.478E-10* | 465.3 | 100 | 0.05998 | *0.0005579* | ***2.923E-06*** |
| ***HexCer d18:1/24:0*** | 3.687 | 0.661 | 3.892 | 0.6089 | 0.9679 | 5.909 | 1.372 | ***2.33E-05*** | *0.000819* | 3.74 | 0.617 | 0.999168 | 0.9876758 | ***6.959E-05*** |
| ***Cer d18:1/18:0*** | 566.6 | 76.71 | 482.6 | 20.6 | 0.722002 | 1051 | 204.4 | ***6.92E-07*** | *6.821E-07* | 680.2 | 216.6 | 0.391567 | 0.102583 | ***0.0001273*** |
| ***Cer d18:1/20:0*** | 223.4 | 19.63 | 205.9 | 26.05 | 0.974883 | 418.5 | 148.8 | ***5.57E-05*** | *0.0001608* | 248.6 | 56.74 | 0.903554 | 0.7596323 | ***0.0006952*** |
| ***Cer d18:1/24:0*** | 212.2 | 55.54 | 188.8 | 33.61 | 0.943512 | 378.8 | 121.5 | ***0.000485*** | *0.0006887* | 236.1 | 82.94 | 0.915511 | 0.6979853 | ***0.0046342*** |
| ***Cer d18:1/16:1*** | 0.152 | 0.033 | 0.1969 | 0.0291 | 0.834313 | 0.4297 | 0.1502 | ***9.29E-06*** | *0.0010656* | 0.262 | 0.134 | 0.113556 | 0.6469517 | ***0.0093566*** |
| ***DHCer d18:0/20:0*** | 4.375 | 0.895 | 4.563 | 0.6221 | 0.996379 | 9.24 | 2.186 | ***2.83E-06*** | *7.149E-05* | 6.689 | 2.308 | *0.025331* | 0.1070066 | ***0.0173866*** |
| ***Hex2Cer d18:1/24:1*** | 1.257 | 0.383 | 1.236 | 0.2515 | 0.999633 | 2.119 | 0.5417 | ***0.000467*** | *0.0022402* | 1.504 | 0.425 | 0.572907 | 0.6309486 | ***0.0214507*** |
| ***HexCer d18:1/24:1*** | 22.37 | 4.296 | 20.49 | 2.452 | 0.9672 | 36.9 | 13.62 | ***0.001869*** | *0.0027953* | 25.5 | 6.2 | 0.822316 | 0.6430657 | ***0.0253676*** |
| ***Cer d18:1/24:1*** | 4.672 | 0.811 | 4.271 | 0.4633 | 0.891258 | 6.583 | 1.399 | ***0.003041*** | *0.0021941* | 5.039 | 1.356 | 0.881375 | 0.5606311 | ***0.0285245*** |
| **Hex2Cer d18:1/18:0** | 0.981 | 0.309 | 1.362 | 0.2195 | 0.379742 | 1.532 | 0.701 | 0.057214 | 0.8971465 | 1.137 | 0.433 | 0.8763 | 0.7911242 | 0.2854244 |
| **Hex2Cer d18:1/24:0** | 1.567 | 0.415 | 1.641 | 0.2521 | 0.985268 | 2.076 | 0.4868 | *0.049571* | 0.2161809 | 1.738 | 0.423 | 0.799625 | 0.9708256 | 0.3289885 |
| **DHHexCer d18:0/26:0** | 1.145 | 0.435 | 1.192 | 0.2889 | 0.995814 | 1.385 | 0.5244 | 0.565459 | 0.8066307 | 1.128 | 0.281 | 0.999693 | 0.9905656 | 0.5476238 |
| **Cer d18:1/22:0** | 70.58 | 18.47 | 101.6 | 12.06 | 0.213518 | 115.8 | 36.91 | *0.01323* | 0.8177353 | 97.3 | 42.21 | 0.23528 | 0.9931654 | 0.5838938 |
| **HexCer d18:1/26:1** | 16.93 | 5.796 | 13.66 | 3.351 | 0.766706 | 18.57 | 10.57 | 0.945631 | 0.5059366 | 15.29 | 3.049 | 0.946071 | 0.9657709 | 0.7224351 |
| **HexCer d18:1/20:1** | 36.16 | 15.71 | 33.83 | 3.829 | 0.972198 | 33.92 | 5.65 | 0.964831 | 0.9999985 | 39.02 | 9.234 | 0.930924 | 0.7888853 | 0.7376812 |
| **DHCer d18:0/24:0** | 6.578 | 2.458 | 4.88 | 1.193 | 0.482798 | 6.881 | 3.097 | 0.991297 | 0.3731204 | 5.765 | 1.672 | 0.862551 | 0.8866952 | 0.7383133 |
| **DHCer d18:0/16:0** | 0.194 | 0.067 | 0.3024 | 0.05219 | 0.355823 | 0.3583 | 0.161 | *0.036044* | 0.8406404 | 0.298 | 0.174 | 0.287263 | 0.9999042 | 0.7519232 |
| **DHHexCer d18:0/16:0** | 5.348 | 1.491 | 4.812 | 0.7978 | 0.876517 | 5.296 | 1.97 | 0.999793 | 0.9145858 | 4.685 | 0.822 | 0.725055 | 0.9982258 | 0.7960293 |
| **Hex2Cer d18:1/16:0** | 0.659 | 0.25 | 0.6672 | 0.1318 | 0.999885 | 0.7403 | 0.3214 | 0.885979 | 0.9448321 | 0.638 | 0.226 | 0.997908 | 0.996251 | 0.8236569 |
| **Hex2Cer d18:1/22:1** | 0.482 | 0.185 | 0.448 | 0.07958 | 0.970183 | 0.4588 | 0.1694 | 0.985759 | 0.9991041 | 0.516 | 0.117 | 0.958944 | 0.828805 | 0.8527074 |
| **Hex2Cer d18:1/20:1** | 3.48 | 1.083 | 3.941 | 1.177 | 0.835841 | 3.496 | 1.103 | 0.999987 | 0.8632089 | 3.87 | 1.001 | 0.853769 | 0.9992901 | 0.8835643 |
| **Hex2Cer d18:1/18:1** | 0.953 | 0.391 | 0.6689 | 0.2115 | 0.395079 | 0.7792 | 0.4502 | 0.689748 | 0.9324482 | 0.664 | 0.236 | 0.277321 | 0.9999936 | 0.8979726 |
| **Hex2Cer d18:1/26:1** | 0.26 | 0.121 | 0.2053 | 0.1003 | 0.687185 | 0.1739 | 0.082 | 0.219262 | 0.9259519 | 0.204 | 0.071 | 0.586252 | 0.9999976 | 0.9080656 |
| **HexCer d18:1/18:0** | 10.18 | 2.838 | 9.865 | 1.711 | 0.993717 | 8.52 | 2.291 | 0.433326 | 0.7198889 | 9.147 | 2.368 | 0.776721 | 0.9424531 | 0.9461707 |
| **HexCer d18:1/22:0** | 5.09 | 0.936 | 5.86 | 0.4787 | 0.51193 | 5.368 | 1.329 | 0.940748 | 0.8259102 | 5.646 | 1.257 | 0.668811 | 0.9821034 | 0.9474773 |
| **HexCer d18:1/22:1** | 11.42 | 2.057 | 11.66 | 1.177 | 0.998389 | 12.78 | 4.512 | 0.734981 | 0.8892538 | 12.08 | 2.697 | 0.958681 | 0.9931693 | 0.957625 |
| **Hex2Cer d18:1/22:0** | 0.662 | 0.214 | 0.8883 | 0.1308 | 0.382568 | 0.8339 | 0.2506 | 0.515763 | 0.9815665 | 0.884 | 0.403 | 0.295278 | 0.9999901 | 0.9799926 |
| **DHHexCer d18:0/22:0** | 4.491 | 1.525 | 4.737 | 0.7301 | 0.985507 | 4.187 | 1.672 | 0.962671 | 0.8786949 | 4.401 | 1.263 | 0.998933 | 0.9681425 | 0.9881735 |
| **DHHexCer d18:0/24:0** | 14.54 | 3.602 | 13.08 | 2.42 | 0.862755 | 15.99 | 4.879 | 0.815246 | 0.4486658 | 15.45 | 2.993 | 0.945026 | 0.6172671 | 0.9893083 |
| **HexCer d18:1/26:0** | 15.05 | 5.379 | 14.54 | 4.974 | 0.996015 | 12.03 | 4.215 | 0.465941 | 0.7270552 | 12.7 | 3.356 | 0.663764 | 0.8700618 | 0.989482 |
| **DHHex2Cer d18:0/20:0** | 2.203 | 0.497 | 2.853 | 0.4936 | 0.300245 | 2.381 | 1.09 | 0.94549 | 0.6024048 | 2.469 | 0.589 | 0.842411 | 0.7424401 | 0.9936761 |
| **HexCer d18:1/18:1** | 16.85 | 5.93 | 19.07 | 1.221 | 0.813411 | 19.87 | 5.165 | 0.53493 | 0.989804 | 19.29 | 4.814 | 0.694602 | 0.9997831 | 0.9944716 |
| **HexCer d18:1/16:0** | 5.702 | 1.871 | 5.037 | 1.142 | 0.849897 | 4.941 | 1.67 | 0.724456 | 0.9994746 | 4.775 | 1.481 | 0.586429 | 0.9897562 | 0.996306 |
| **DHCer d18:0/18:0** | 16.46 | 6.037 | 17.35 | 2.209 | 0.984813 | 16.31 | 4.334 | 0.999876 | 0.9782733 | 16.8 | 5.281 | 0.998741 | 0.9966764 | 0.9966257 |
| **DHHexCer d18:0/20:0** | 1.411 | 0.333 | 1.45 | 0.3818 | 0.998445 | 1.51 | 0.7273 | 0.965191 | 0.9947767 | 1.465 | 0.305 | 0.993939 | 0.9999094 | 0.9969819 |
| **DHHex2Cer d18:0/18:0** | 0.1 | 0.042 | 0.09924 | 0.04872 | 0.99999 | 0.0846 | 0.0444 | 0.842509 | 0.9061018 | 0.088 | 0.03 | 0.921168 | 0.9562339 | 0.9977374 |
| **HexCer d18:1/20:0** | 9.226 | 4.042 | 9.533 | 2.356 | 0.997127 | 7.881 | 2.28 | 0.757101 | 0.7304997 | 8.072 | 2.539 | 0.83122 | 0.7969803 | 0.999122 |
| **DHHexCer d18:0/18:0** | 3.279 | 0.807 | 3.124 | 0.4187 | 0.976224 | 3.214 | 0.8565 | 0.997362 | 0.9956153 | 3.174 | 0.692 | 0.989128 | 0.9992306 | 0.9994587 |
| **Cer d18:1/22:1** | 2.191 | 0.398 | 2.268 | 0.2903 | 0.993563 | 2.462 | 0.8121 | 0.724636 | 0.9186086 | 2.477 | 0.615 | 0.689844 | 0.9004961 | 0.999935 |
| **Cer d18:1/20:1** | 36.09 | 11.88 | 27.27 | 3.04 | 0.302914 | 36.41 | 11.3 | 0.999855 | 0.3044595 | 36.63 | 7.79 | 0.999326 | 0.2850502 | 0.999962 |
| **DHCer d18:0/22:0** | 9.898 | 3.343 | 9.677 | 1.91 | 0.999293 | 10.27 | 4.656 | 0.995377 | 0.9884655 | 10.33 | 3.039 | 0.992301 | 0.9840453 | 0.9999724 |
| **Cer d18:1/26:1** | 2.258 | 0.851 | 1.929 | 0.3882 | 0.723348 | 2.039 | 0.3387 | 0.860163 | 0.9864444 | 2.051 | 0.612 | 0.878815 | 0.981743 | 0.9999748 |
| **HexCer d18:1/16:1** | 0.377 | 0.049 | 0.3901 | 0.02727 | 0.988542 | 0.3767 | 0.1118 | 1 | 0.9901417 | 0.378 | 0.107 | 0.99997 | 0.9929591 | 0.9999818 |
| **Cer d18:1/26:0** | 1.856 | 0.642 | 1.599 | 0.3382 | 0.755107 | 1.579 | 0.5317 | 0.624724 | 0.9998286 | 1.578 | 0.369 | 0.622182 | 0.9998042 | 1 |

Concentrations of the 46 detected ceramides from WT+Vehicle, PS19+Vehicle, and PS19+PDDC treated cortical tissue. SD=Standard Deviation. Red indicate statistically significant ceramides.

**Table S5. Ceramide values and *P* values from AAV-hTau mice.**

|  | **Vehicle Hippocampus** | | **PDDC Hippocampus** | | | **Vehicle Cortex** | | | **PDDC Cortex** | | | |
| --- | --- | --- | --- | --- | --- | --- | --- | --- | --- | --- | --- | --- |
| **Ceramides** | **ng/mg** | **SD** | **ng/mg** | **SD** | ***P* value vs Veh Hip** | **ng/mg** | **SD** | ***P* value vs Veh Hip** | **ng/mg** | **SD** | ***P* value vs Veh Hip** | ***P* value vs Veh Ctx** |
| **Cer d18:1/18:0** | 1334 | 115.5 | 635.1 | 205.2 | ***8.464E-08*** | 741.8 | 240.1 | ***5.489E-06*** | 641.3 | 197.1 | *1.02E-07* | 0.7019956 |
| **Cer d18:1/24:1** | 7.49 | 0.756 | 4.684 | 0.9734 | ***1.419E-05*** | 5.208 | 1.224 | ***0.0006016*** | 5.342 | 1.118 | *0.000665* | 0.9926713 |
| **Cer d18:1/18:1** | 34.55 | 8.496 | 17.51 | 8.891 | ***0.0014138*** | 21.34 | 9.872 | ***0.0242447*** | 17.15 | 7.831 | *0.001111* | 0.7445908 |
| **Cer d18:1/16:0** | 809.2 | 249.9 | 424.2 | 219.5 | ***0.0042584*** | 505.2 | 253.1 | ***0.0433151*** | 369.1 | 156.5 | *0.001002* | 0.56504 |
| **Cer d18:1/24:0** | 407.4 | 83.02 | 270.8 | 124.1 | 0.0887809 | 300.5 | 163.7 | 0.2853296 | 253 | 88.16 | *0.044384* | 0.8304928 |
| **HexCer d18:1/22:1** | 12.77 | 1.24 | 11.03 | 1.756 | 0.18515 | 12.41 | 2.596 | 0.9769059 | 12.84 | 1.303 | 0.9998276 | 0.9569903 |
| **HexCer d18:1/20:1** | 41.74 | 10.22 | 32.9 | 4.505 | 0.3291983 | 41.87 | 18.84 | 0.999995 | 36.62 | 6.188 | 0.7521425 | 0.7378143 |
| **HexCer d18:1/24:1** | 26.3 | 2.81 | 22.22 | 3.904 | 0.339757 | 26.66 | 6.103 | 0.998947 | 25.43 | 6.37 | 0.9835366 | 0.4959817 |
| **HexCer d18:1/16:1** | 0.4454 | 0.06012 | 0.4066 | 0.06492 | 0.4986336 | 0.45 | 0.0523 | 0.9985584 | 0.4114 | 0.05202 | 0.6046525 | 0.5024841 |
| **Cer d18:1/22:1** | 2.459 | 0.2854 | 2.281 | 0.3246 | 0.728715 | 2.327 | 0.4575 | 0.8846835 | 2.495 | 0.3665 | 0.9966913 | 0.7629022 |
| **Cer d18:1/20:0** | 292.5 | 63.6 | 247.9 | 102.9 | 0.7566504 | 304.9 | 127.1 | 0.9936931 | 262.7 | 76.41 | 0.9106036 | 0.7857472 |
| **DHHex2Cer d18:0/18:0** | 0.1042 | 0.02457 | 0.08685 | 0.04188 | 0.787557 | 0.1122 | 0.0355 | 0.9767794 | 0.1008 | 0.04784 | 0.9977009 | 0.9267124 |
| **Hex2Cer d18:1/20:1** | 4.187 | 0.9917 | 3.75 | 1.286 | 0.8260041 | 3.155 | 0.7996 | 0.2383366 | 4.523 | 1.086 | 0.9111508 | 0.0523952 |
| **HexCer d18:1/26:1** | 16.4 | 2.879 | 14.18 | 6.052 | 0.8355199 | 16.75 | 8.469 | 0.9992593 | 16.76 | 3.413 | 0.9990495 | 1 |
| **DHHex2Cer d18:0/20:0** | 2.468 | 0.4412 | 2.258 | 0.4081 | 0.8688056 | 2.638 | 0.6669 | 0.9352146 | 2.453 | 0.7269 | 0.9999378 | 0.9060335 |
| **DHHexCer d18:0/26:0** | 1.207 | 0.2035 | 1.083 | 0.3873 | 0.8815037 | 1.155 | 0.4466 | 0.9913071 | 1.292 | 0.3374 | 0.9585096 | 0.851154 |
| **Cer d18:1/26:1** | 2.301 | 0.3409 | 2.044 | 0.8291 | 0.8847457 | 2.374 | 1.164 | 0.997244 | 2.294 | 0.355 | 0.999998 | 0.9959055 |
| **DHCer d18:0/16:0** | 0.2944 | 0.04187 | 0.2648 | 0.07052 | 0.9039078 | 0.2784 | 0.0887 | 0.9850364 | 0.3012 | 0.1319 | 0.9985876 | 0.9521987 |
| **Hex2Cer d18:1/18:0** | 1.349 | 0.2197 | 1.257 | 0.2634 | 0.9209681 | 1.314 | 0.2701 | 0.995578 | 1.348 | 0.4118 | 1 | 0.995033 |
| **Hex2Cer d18:1/16:0** | 0.7063 | 0.1271 | 0.7667 | 0.2322 | 0.930236 | 0.6593 | 0.1457 | 0.9701356 | 0.7823 | 0.2745 | 0.8719934 | 0.613896 |
| **DHCer d18:0/22:0** | 10.26 | 1.945 | 11.07 | 3.055 | 0.943664 | 11.32 | 3.247 | 0.901275 | 9.777 | 3.631 | 0.9873727 | 0.7192503 |
| **DHCer d18:0/20:0** | 6.07 | 1.619 | 5.488 | 2.403 | 0.9468695 | 6.041 | 2.717 | 0.9999937 | 5.686 | 2.099 | 0.9837275 | 0.9870355 |
| **Hex2Cer d18:1/18:1** | 0.7841 | 0.1449 | 0.8739 | 0.4125 | 0.952977 | 1.046 | 0.4987 | 0.4795724 | 0.7951 | 0.2936 | 0.999905 | 0.4709288 |
| **DHHexCer d18:0/20:0** | 1.46 | 0.21 | 1.55 | 0.3899 | 0.9539473 | 1.459 | 0.3107 | 1 | 1.589 | 0.4634 | 0.8769753 | 0.8765033 |
| **DHHexCer d18:0/22:0** | 4.724 | 0.8835 | 5.014 | 1.523 | 0.9749295 | 5.361 | 1.365 | 0.8194543 | 4.694 | 1.798 | 0.9999713 | 0.772035 |
| **HexCer d18:1/22:0** | 5.918 | 0.7566 | 5.736 | 0.94 | 0.978621 | 6.03 | 0.991 | 0.9955845 | 5.835 | 1.122 | 0.9978743 | 0.9740481 |
| **Cer d18:1/20:1** | 36.69 | 4.878 | 34.92 | 10.27 | 0.9811448 | 39.3 | 14.59 | 0.9510248 | 33.14 | 7.434 | 0.8716473 | 0.5577605 |
| **HexCer d18:1/20:0** | 9.632 | 2.405 | 10.19 | 3.011 | 0.9819715 | 12.14 | 3.393 | 0.3928839 | 8.998 | 3.525 | 0.9735785 | 0.1707432 |
| **HexCer d18:1/18:0** | 10.6 | 2.047 | 10.16 | 2.415 | 0.9842803 | 11.8 | 2.022 | 0.7887489 | 10.21 | 3.392 | 0.9886627 | 0.5703363 |
| **HexCer d18:1/26:0** | 13.87 | 2.338 | 14.63 | 5.006 | 0.9867791 | 14.4 | 5.445 | 0.9959655 | 15.76 | 5.446 | 0.839701 | 0.9330385 |
| **Cer d18:1/16:1** | 0.2578 | 0.06298 | 0.2437 | 0.1104 | 0.9929048 | 0.2494 | 0.1288 | 0.9987048 | 0.2472 | 0.1221 | 0.9969355 | 0.9999708 |
| **Hex2Cer d18:1/26:1** | 0.2388 | 0.05464 | 0.2263 | 0.1023 | 0.9935549 | 0.2466 | 0.07865 | 0.998595 | 0.244 | 0.134 | 0.9994961 | 0.9999419 |
| **Hex2Cer d18:1/24:0** | 1.895 | 0.2541 | 1.836 | 0.5593 | 0.9947247 | 1.883 | 0.6469 | 0.9999588 | 1.907 | 0.4934 | 0.9999559 | 0.9996319 |
| **HexCer d18:1/24:0** | 4.462 | 0.573 | 4.323 | 1.393 | 0.9952301 | 4.755 | 1.762 | 0.9643472 | 4.432 | 0.9008 | 0.9999479 | 0.9454951 |
| **DHHexCer d18:0/24:0** | 14.94 | 1.899 | 15.3 | 3.422 | 0.9961126 | 15.06 | 3.966 | 0.9998828 | 15.77 | 3.771 | 0.9558105 | 0.9712101 |
| **HexCer d18:1/18:1** | 19.76 | 2.527 | 19.4 | 3.077 | 0.9978644 | 19.6 | 7.086 | 0.9998217 | 19.93 | 3.397 | 0.9998171 | 0.9984484 |
| **DHHexCer d18:0/16:0** | 5.223 | 0.8055 | 5.339 | 1.471 | 0.9979349 | 5.785 | 1.404 | 0.843734 | 5.427 | 1.565 | 0.9889653 | 0.9455445 |
| **DHCer d18:0/24:0** | 6.277 | 1.318 | 6.441 | 2.305 | 0.9987075 | 6.962 | 2.653 | 0.9289846 | 6.13 | 2.432 | 0.9990398 | 0.8636738 |
| **Cer d18:1/22:0** | 101.1 | 16.57 | 103.1 | 41.69 | 0.9993531 | 100.1 | 42.88 | 0.9999194 | 100.4 | 26.64 | 0.9999609 | 0.9999984 |
| **HexCer d18:1/16:0** | 5.713 | 1.313 | 5.809 | 1.647 | 0.9994466 | 6.589 | 1.53 | 0.7537671 | 5.522 | 2.273 | 0.9956648 | 0.5836951 |
| **Hex2Cer d18:1/22:0** | 0.9032 | 0.1551 | 0.8901 | 0.2966 | 0.999518 | 0.8595 | 0.2474 | 0.9854328 | 0.888 | 0.2695 | 0.9992506 | 0.9951457 |
| **DHHexCer d18:0/18:0** | 3.445 | 0.439 | 3.483 | 0.87 | 0.9996321 | 3.626 | 0.8162 | 0.9679516 | 3.529 | 0.9072 | 0.9959705 | 0.9939221 |
| **Hex2Cer d18:1/24:1** | 1.603 | 0.3286 | 1.586 | 0.5667 | 0.9999037 | 1.675 | 0.7706 | 0.9942502 | 1.59 | 0.5241 | 0.9999523 | 0.988808 |
| **DHCer d18:0/18:0** | 18.45 | 2.65 | 18.36 | 4.663 | 0.9999736 | 19.26 | 6.619 | 0.9851346 | 18.14 | 3.945 | 0.9989526 | 0.9561019 |
| **Cer d18:1/26:0** | 1.834 | 0.347 | 1.825 | 0.5974 | 0.9999874 | 1.969 | 0.5953 | 0.9681499 | 1.81 | 0.7307 | 0.9997961 | 0.9425715 |
| **Hex2Cer d18:1/22:1** | 0.4901 | 0.07467 | 0.4886 | 0.1928 | 0.9999974 | 0.5563 | 0.2389 | 0.8394496 | 0.4727 | 0.06711 | 0.9956601 | 0.6890501 |

Concentrations of the 46 detected ceramides from Vehicle Hippocampus, PDDC hippocampus, Vehicle cortex, and PDDC cortex tissue. SD=Standard Deviation. Red indicate statistically significant ceramides.


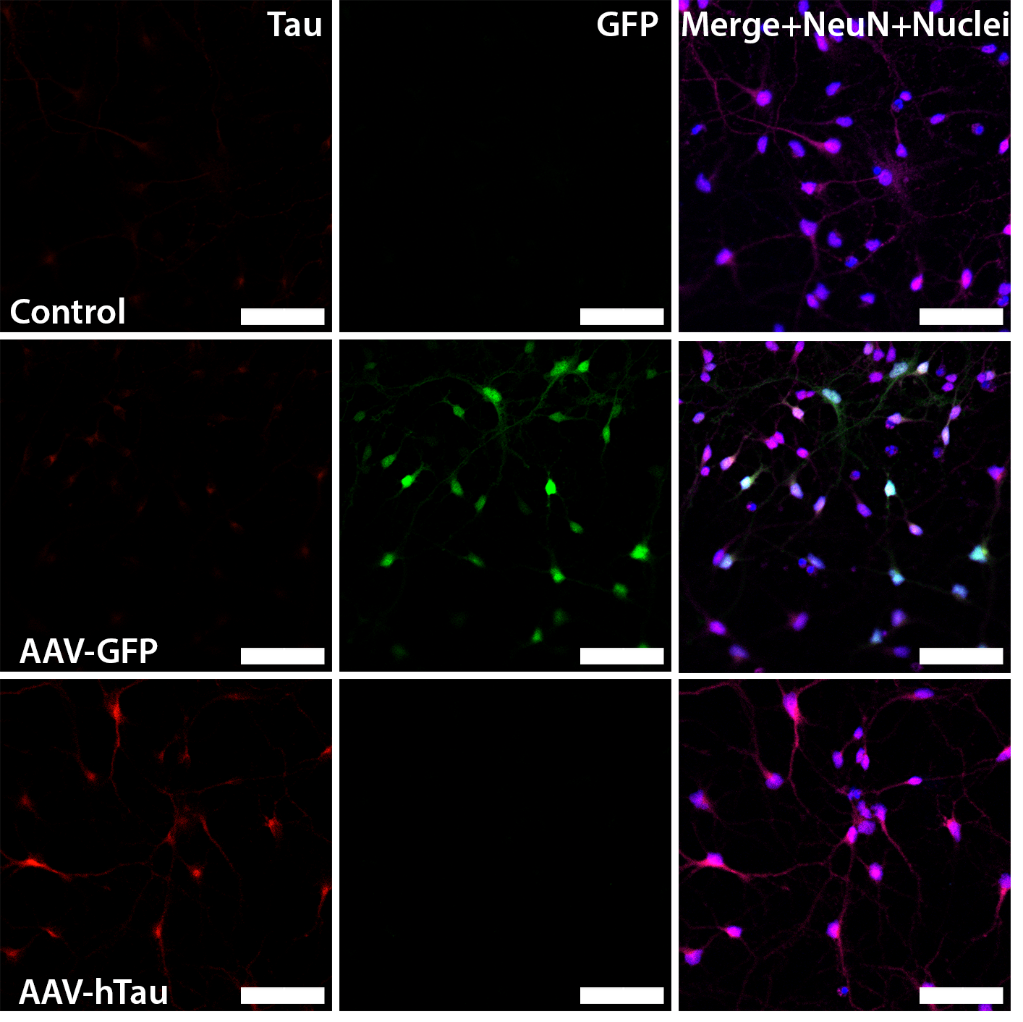


**Fig. S1. AAV-GFP and AAV-hTau infected cells express appropriate proteins.** Representative images of uninfected control (top), AAV-GFP infected (middle), and AAV-hTau(P301L/S320F) infected (bottom) primary rat hippocampal neurons. Cells were stained for pThr181-Tau (left column; red), GFP (middle column; green) and NeuN and nuclei (right column; magenta and blue, respectively). Scale bar, 50 µm.


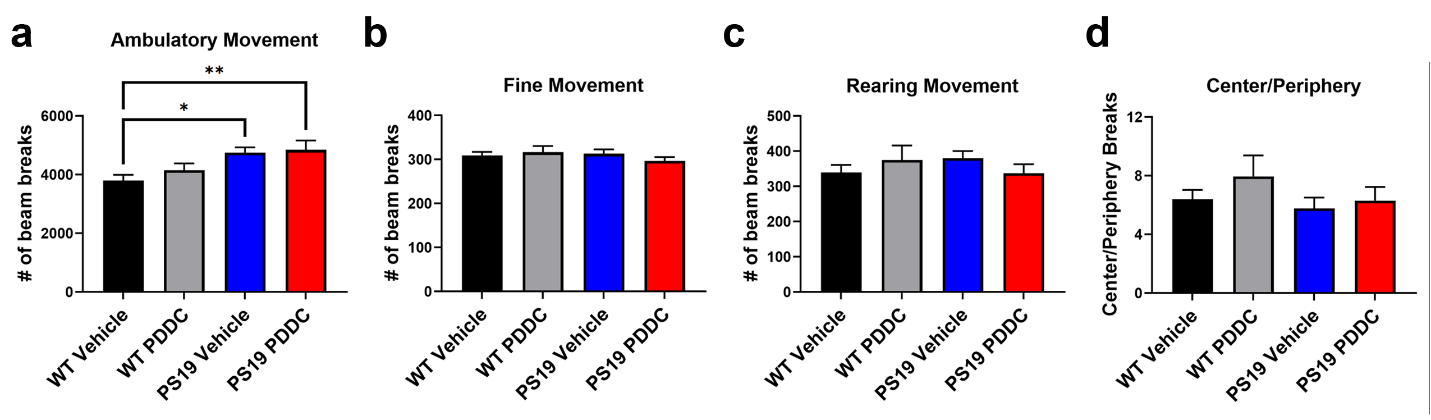


**Fig. S2. PDDC treatment does not negatively affect behavior in WT or PS19 mice. a** Total ambulatory movement is increased in PS19 mice but not affected by PDDC treatment. **b** Fine movement grooming behavior is not affected in any group. **c** Rearing movement is not affected in any group. **d** Ratio of center vs periphery movement is unaffected in all groups. Bars represent mean ± SEM. * *P*<0.05; ** *P* <0.01. n=12-24.


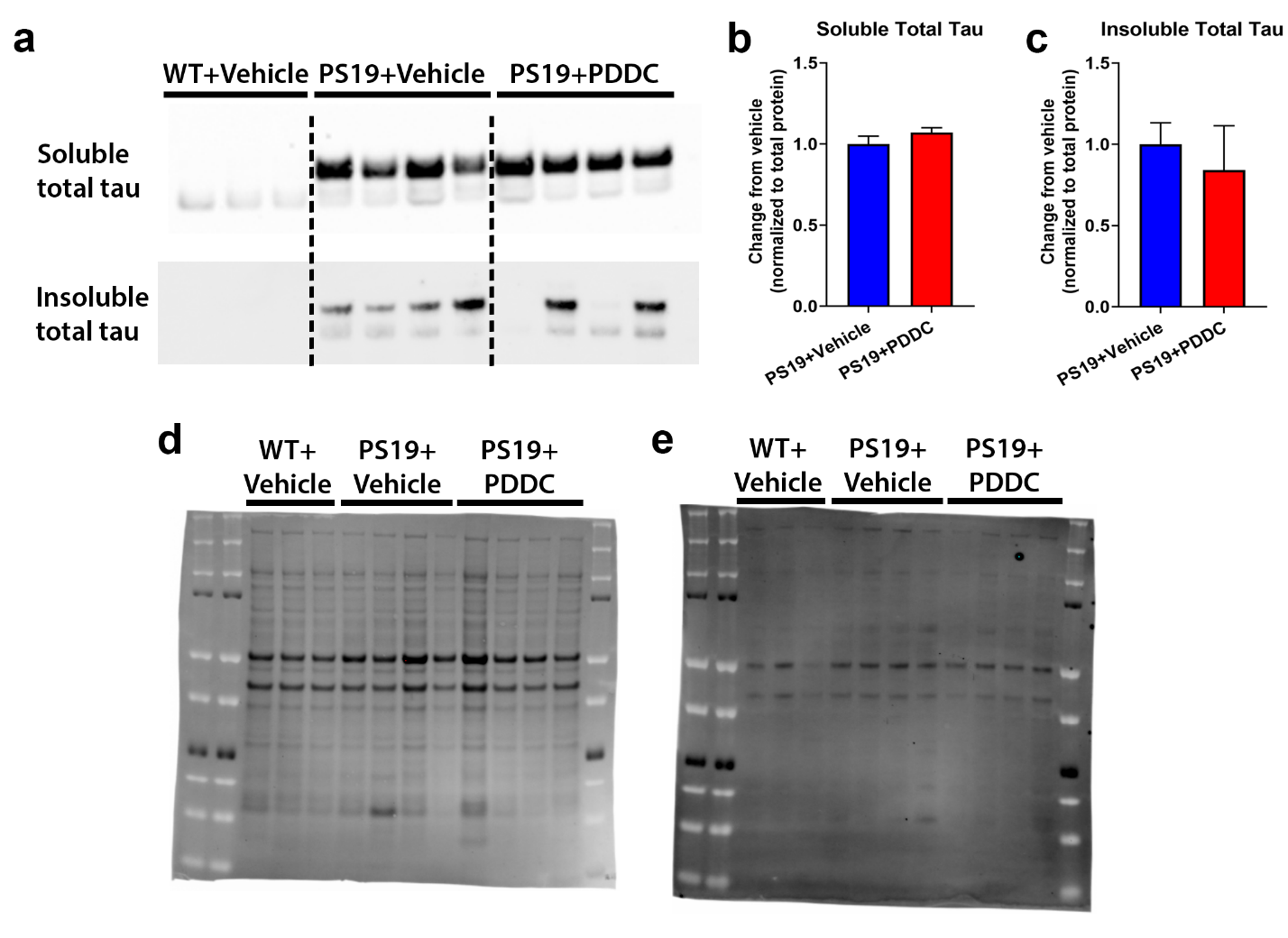


**Fig. S3. PDDC does not alter Sarkosyl-soluble and -insoluble tau fractions. a**) Representative Western blots of total tau expression in the Sarkosyl soluble (top) and insoluble (lower) fractions. **b-c**) Quantification of Western blot total tau expression from the Sarkosyl soluble (**b**) and insoluble (**c**) fractions. Bars represent mean ± SEM. n=6-8. **d-e**) Total protein staining of the Sarkosyl soluble (**d**) and insoluble (**e**) fraction.


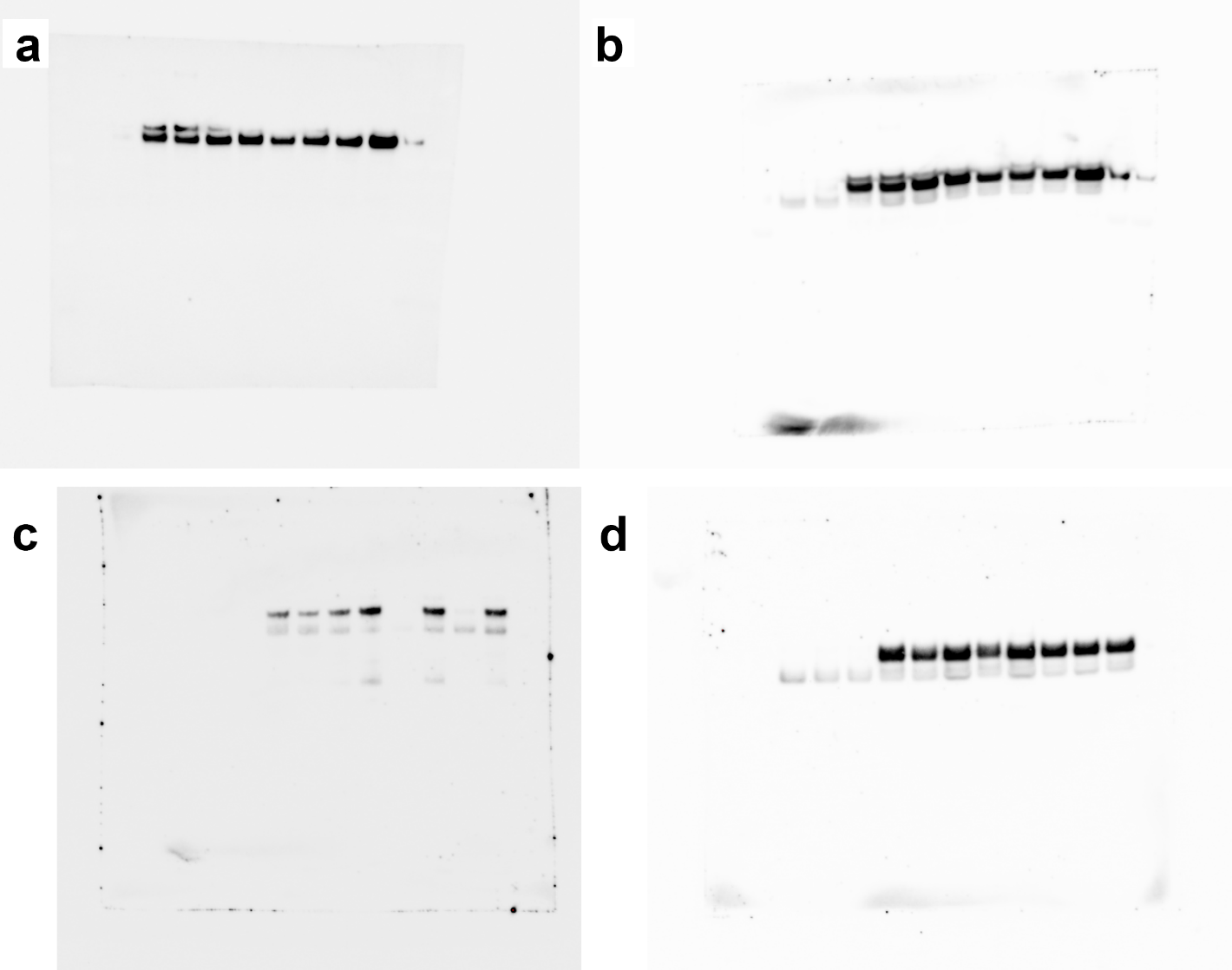


**Fig. S4. Uncropped western blot images.** Complete blots of pThr181-Tau (**a**), Total tau (**b**), Sarkosyl insoluble tau (**c**), and Sarkosyl insoluble tau (**d**).


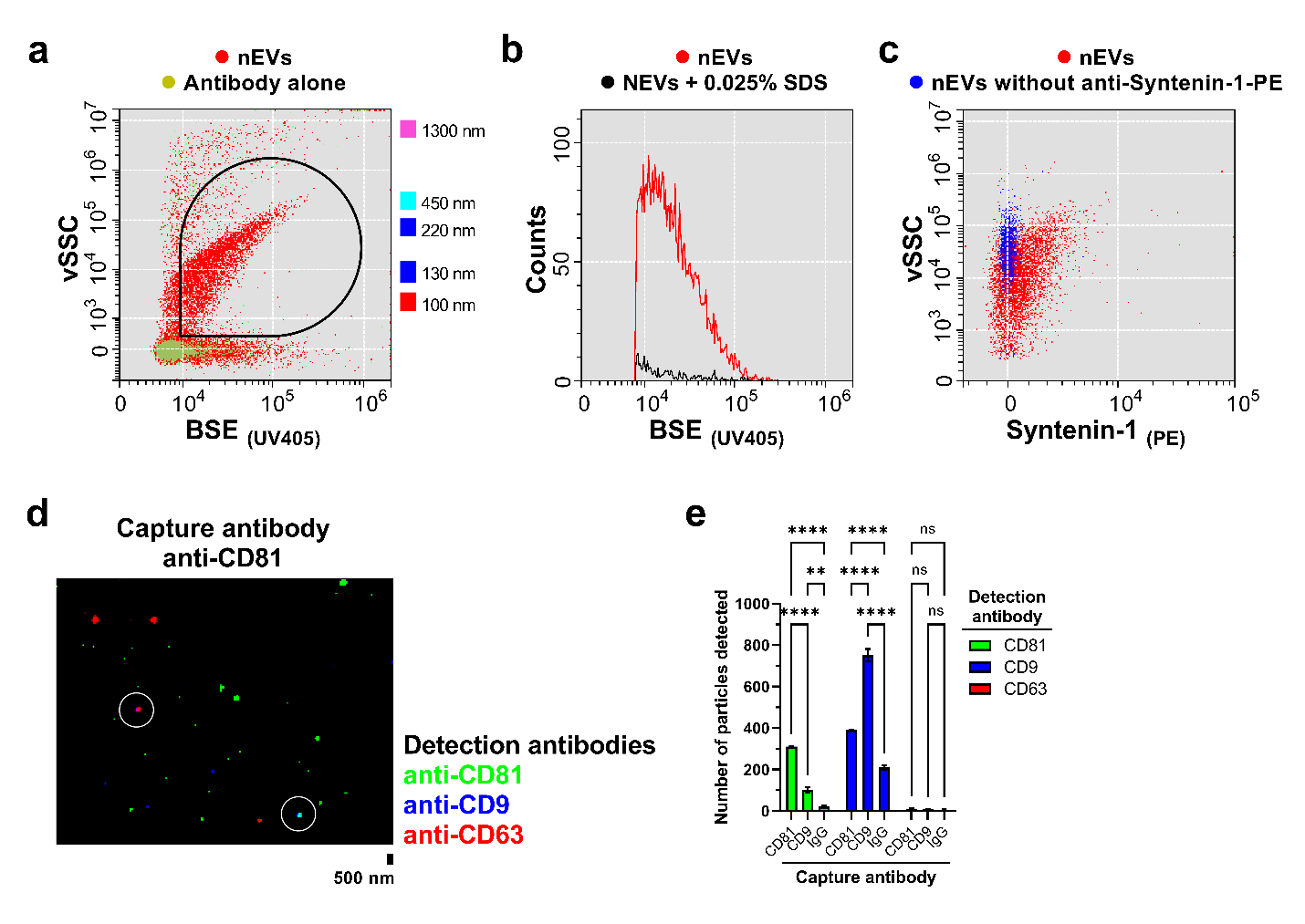


**Fig. S5.** **Characterization of L1CAM+ nEVs immunoprecipitated from mouse plasma.** **a–c)** High-sensitivity nanoscale multiplex flow cytometry analysis (FCA) of partially permeabilized nEVs isolated from the pooled plasma of P301S mice (n=4) via L1CAM immunocapture. FCA was based on the detection of the violet size scatter (vSSC), as an indicator of nanoparticle size, in function of the fluorescent signal of nEVs co-labeled with the fluorescent EV marker blue succinimidyl ester (BSE) and PE-tagged anti-Syntenin-1 antibody. In **a**, a dot plot shows the violet size scatter (vSSC) in function of the BSE signal of nEVs (red events), with BSE+ events enclosed by a gate (black line) designated based on the background signal of mixed BSE and antibody alone (yellow events). A color-coded size range based on the vSSC of FITC-tagged beads is included on the right for the size comparison of events. In **b**, a histogram shows the signal sensitivity of BSE-gated events (red line) to treatment with SDS detergent (black line) confirming the membranous composition of detected events. In **c**, a dot plot shows the vSSC vs. PE signal of BSE-gated events in L1CAM+ nEVs labeled with the PE-tagged EV marker anti-Syntenin-1 (red events) or unlabeled (blue events). Similar results were obtained in pooled L1CAM+ nEVs immunocaptured from the plasma of WT mice (n=6; data not shown). **d)** ExoView® image showing the fluorescence detection of nanoparticles (under the upper limit of 500 nm based on the scale bar) captured using anti-CD81 antibody, absorbed on the ExoView® chip surface, and detected by a cocktail of fluorescent anti-CD9-488 (blue), anti-CD81-555 (green) and anti-CD63-647 (red) antibodies. The detection of tetraspanins in captured CD81+ EVs is based on the fluorophores detected, with some particles showing a single fluorescent signal above the detection threshold, or multiple fluorescent signals (circled particles). **e)** A bar graph shows the number of tetraspanin-positive EVs (captured by anti-CD81 and CD9 antibodies and detected with either anti-CD9, -CD63 or -CD81 fluorescent antibodies) in the L1CAM IP eluate, as detected by ExoView®. The signal from particles captured with a hamster and rat isotype control (IgG) accounts for the non-specific binding of EVs to the ExoView® chip. Bars represent the mean ± SEM. ** *P*<0.01; **** *P*<0.0001. Statistical analysis: two-way ANOVA with Fisher’s LSD test of multiple comparisons.


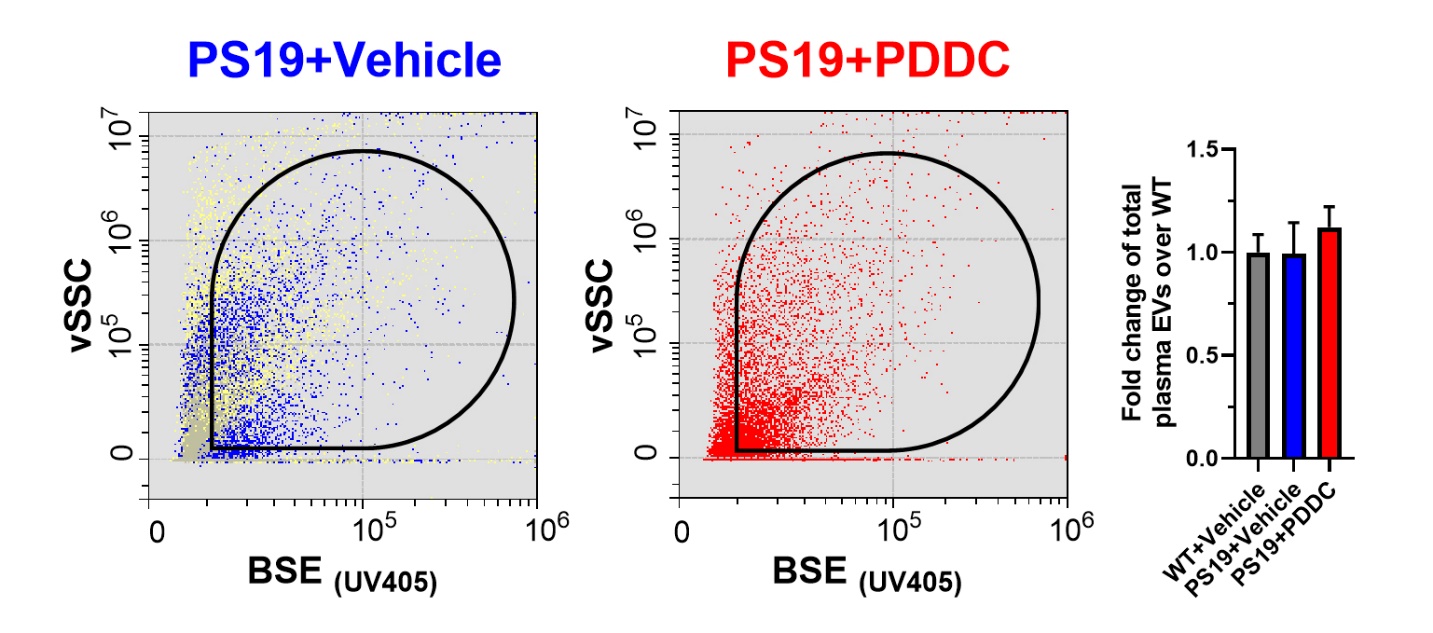


**Fig. S6. PDDC does not affect total EVs by FCA analysis.** Representative dot plots show the vSSC vs. BSE signal of plasma EVs from a vehicle- (left, blue events) and PDDC-treated (middle, red events) PS19 mice. Black line: BSE+ event gating. Bar graph: fold change of BSE+ events (expressed as a percentage of total events) over WT (right).


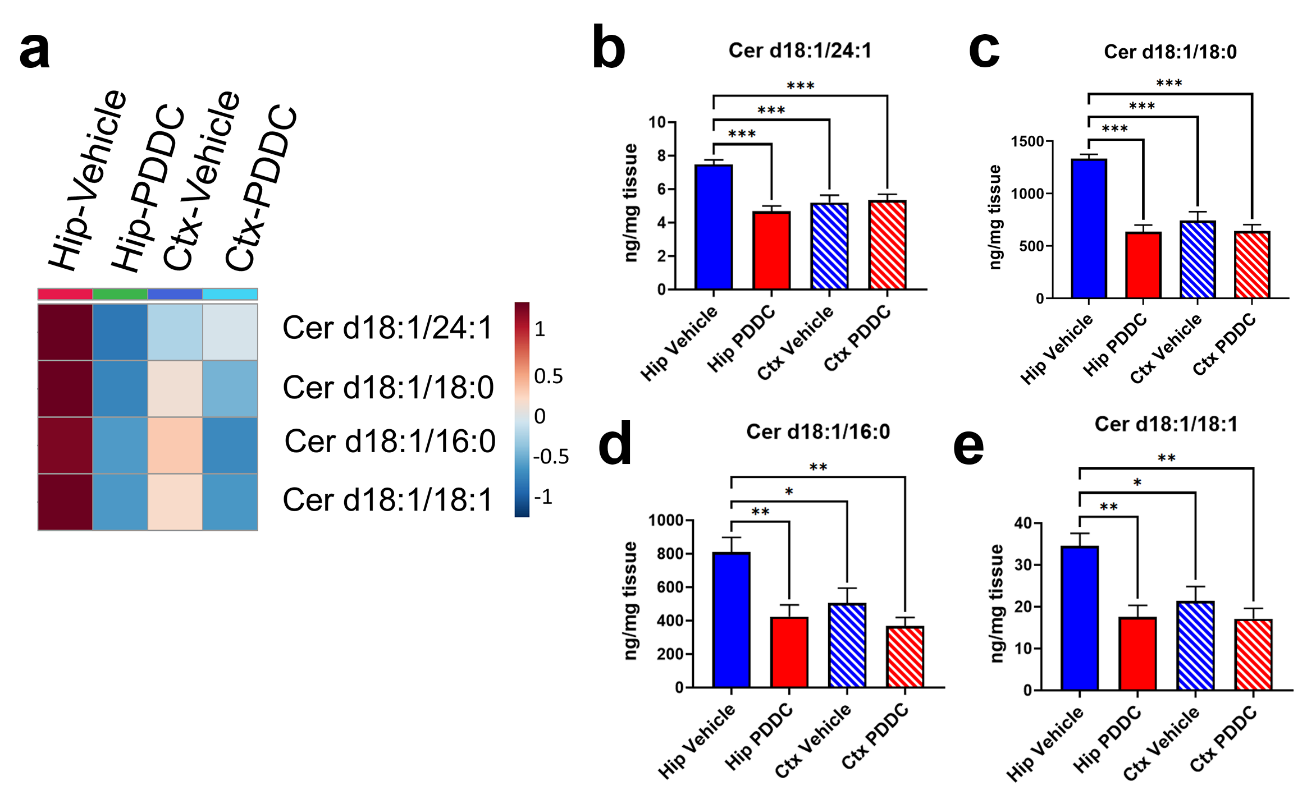
**Fig. S7. PDDC reduces ceramide levels in the hippocampus of AAV-hTau mice.** **a**) Heatmap showing the significantly reduced ceramides in PDDC- vs vehicle-treated PS19 mice. Colors represent relative abundance of each ceramide. **b-e**) Ceramide levels in the hippocampus and cortex of AAV-hTau mice treated with vehicle or PDDC. One-way ANOVA with Tukey’s multiple comparison. Bars represent mean±SEM. * *P*<0.05, ** *P*<0.01, *** *P*<0.001.

**SUPPLEMENTARY REFERENCES**

1. H. Masuya *et al.*, Implementation of the modified-SHIRPA protocol for screening of dominant phenotypes in a large-scale ENU mutagenesis program. *Mammalian Genome* **16**, 829-837 (2005).

2. D. C. Rogers *et al.*, Use of SHIRPA and discriminant analysis to characterise marked differences in the behavioural phenotype of six inbred mouse strains. *Behavioural Brain Research* **105**, 207-217 (1999).
